# Protecting organs-at-risk in cancer therapies through temporary organ displacement: a comprehensive review

**DOI:** 10.3389/or.2025.1655365

**Published:** 2025-09-05

**Authors:** Rance B. Tino, Michael Li, Amirreza Heshmat, Ayush Suresh, AnaSimone Guillaume, Kristy K. Brock, Bruno C. Odisio, Eugene J. Koay

**Affiliations:** ^1^ Department of GI Radiation Oncology, The University of Texas MD Anderson Cancer Center, Houston, TX, United States; ^2^ Department of Imaging Physics, The University of Texas MD Anderson Cancer Center, Houston, TX, United States; ^3^ Department of Interventional Radiology, The University of Texas MD Anderson Cancer Center, Houston, TX, United States

**Keywords:** organ sparing, radiation therapy, temporary organ displacement, organs-at-risk, dose toxicity, thermal ablation, non-target organ injury, spacers

## Abstract

Radiation therapy (RT) and locoregional ablation are cornerstones of modern oncology, yet their therapeutic potential is frequently limited by the challenge of sparing healthy organs-at-risk (OARs) from treatment-related complications. Temporary organ displacement (TOD) techniques directly address this issue by creating a physical separation using ‘spacers’ during treatment, thereby minimizing collateral damage while enhancing therapeutic precision. The clinical benefits, including improved tumor control, reduced morbidity, and enhanced survival, are documented across malignancies of the head and neck, thorax, abdomen, and pelvis. To create a unified framework for this evolving field, this comprehensive review provides a systematic classification of TOD techniques based on invasiveness, administration, device technology and the accompanying treatment mo`dality. Furthermore, we synthesize key historical and recent innovations, from non-invasive maneuvers to advanced surgical spacers, to contextualize current practices. Finally, we address barriers to standardization and highlight emerging concepts such as meta-materials, computational modeling, and digital twins, which provide promising avenues for enhancing personalized cancer care and patient outcomes.

## 1 Introduction

Radiation toxicity and thermal injuries continue to pose significant clinical challenges in the management of cancer when using radiation therapy (RT) and locoregional ablation such as microwave ablation (MWA), radiofrequency ablation (RFA), and cryoablation (CA). Although these treatments are effective in targeting tumors, they can inadvertently damage adjacent organs-at-risk (OARs), resulting to acute and chronic complications that adversely affect the patient’s quality of life. Radiation toxicities arising from RT may present as mucositis, dermatitis, pneumonitis, and reduced blood cell counts (1). In contrast, locoregional ablations can cause pain, bleeding, abscess, and infection due to thermal damage or perforation of OARs involving the gastrointestinal (GI) tract, gallbladder, bile ducts, blood vessels, nerves, diaphragm, kidneys, or ureters (2–4). The severity of these toxicities is influenced by factors such as the total radiation dose or ablation power and duration, the extent of treatment margins, and the proximity of the tumor to OARs. To overcome these challenges, several strategies were established to enhance the safety and effectiveness of treatment. These approaches include the use of optimized fractionation schedules to minimize dose toxicities (5–7), the application of advanced stereotactic imaging and simulations for more precise tumor targeting and OAR delineation (6, 8–10), and the administration of protective agents to minimize toxicities (11–13).

As cancer treatment technologies became increasingly complex, driven by advanced imaging guidance and multi-modal therapies, temporary organ displacement (TOD) remains a simple yet often overlooked concept for protecting OARs. In the clinic, TOD techniques aim to physically separate adjacent OARs from treatment regions using a ‘spacer’ to avoid dose toxicities or injuries. Over time, a range of spacers were introduced to complement RT and locoregional ablation therapies, either by using the patient’s native tissues, repurposing existing implants or by developing new materials, ranging from liquid to solid forms, to improve local tumor control and overall survival (OS) (14–16). More specifically, advances in TOD techniques have led to enhanced effectiveness in maximizing treatment outcomes for numerous cancers involving the prostate (15, 17–19), pelvic (20), vagina (21), pancreas (22), liver (23–28), lung (29–31), peripheral nerves (32), and the head and neck (HNC) (33–37).

Early studies demonstrated the successful clinical translation of spacers, proving their safety, efficacy, and tolerability. These studies also showed that spacers effectively reduced acute side effects after prostate and cervical RT (38–40) and locoregional thermal ablation of the liver, kidney, lung, and bone (24, 28). Tang et al. (2018) provided a systematic review of spacers and their clinical implementation in RT (14), while Garnon et al. (2019) comprehensively review adjunctive thermoprotection strategies, specifically outlining techniques and materials designed to safeguard OARs during thermal ablation (24). Subsequently, reviews by Vaggers et al. (2021) (41) and Lapuz et al. (2024) (42) specifically addressed the use of hydrogel-based spacers in patients with prostate and gynecological cancers receiving brachytherapy (BT), respectively. Building on existing literature, our review provides a comprehensive and systematic classification of both historical and contemporary TOD techniques, detailing their invasiveness, modes of delivery, clinical applications, and device technologies. We synthesize key technical developments and critically assess the factors influencing their successful integration into radiotherapy and locoregional ablation workflows. In addition, we address current limitations in standardization and clinical adoption and explore promising opportunities for next-generation TOD solutions. Special attention is given to emerging trends such as personalized spacer design, advanced customization, and the integration of digital twin (DT) platforms, which hold the potential to further enhance precision and patient outcomes in cancer treatment.

## 2 A brief history of TOD in cancer therapy

In the late 1950s, awareness grew towards preserving ovarian function in patients with genitourinary (GU) cancers during RT (43–45). Notably, Batten and Brown’s clinical report in 1956 described an early form of organ displacement, using lead shells to shield ovaries from the radiation field (46). This principle was extended over the following decades; for instance, Steckel et al. demonstrated the use of catheter balloon occlusions in 1974 to protect against radiation-induced organ damage (47). Later, the introduction of adjunctive RT to treat colorectal adenocarcinomas led to complications such as radiation enteritis, prompting pre-operative RT involving abdominoperineal resection (48–50). In 1980, Gunderson et al. introduced the concept of ‘*Operative displacement*’ showcasing various techniques to protect OARs during post-operative RT, utilizing surgical techniques to displace and immobilize OARs (51). In 1985, surgical organ displacement methods such as ‘*omental envelope*’ (also known as ‘*omental pedicle flap*’ or ‘*omental sling*’) and retroverting the uterus were introduced, to protect the small intestines during pelvic radiation or to avoid potential risk of infertility, respectively (51–53).

At the same time, the proliferation of thermal ablation therapies in interventional radiology (IR) created the urgent need to protect OARs. Consequently, hydrodissection was widely adopted as a protective measure to prevent complications like necrosis or perforation by creating a fluid buffer that physically displaces healthy tissues from the ablation zone. This technique remains a cornerstone of safety in modern RFA, MWA, and CA procedures. Taking inspiration from lens-cortex separation (54) in the early 1980s, modern hydrodissection procedures in IR involves the injection of saline and takes advantage of spaces between the liver or kidney and diaphragm, body wall, bowel, pancreas or stomach. In the early 2000s, hydrodissection was shown to prevent nerve and bowel damage during the RFA of renal tumors (12, 55). When hydrodissection proves insufficient, other techniques are employed, including gas insufflation (56), balloon interposition (57) or probe manipulation (58). While both RT and thermal ablation use displacement techniques like balloons, their applications differ significantly. Organ displacement for image-guided thermal ablation is typically a one-time maneuver performed during the procedure, whereas in RT, the displacement must be reliably reproduced over several days for both treatment simulation and delivery (57, 59). Over time, technological innovations have enhanced material biocompatibility and patient safety, expanding the use of TOD techniques to various cancer types and the conception of more sophisticated spacers.

## 3 Classifications of TOD

**FIGURE 1:**
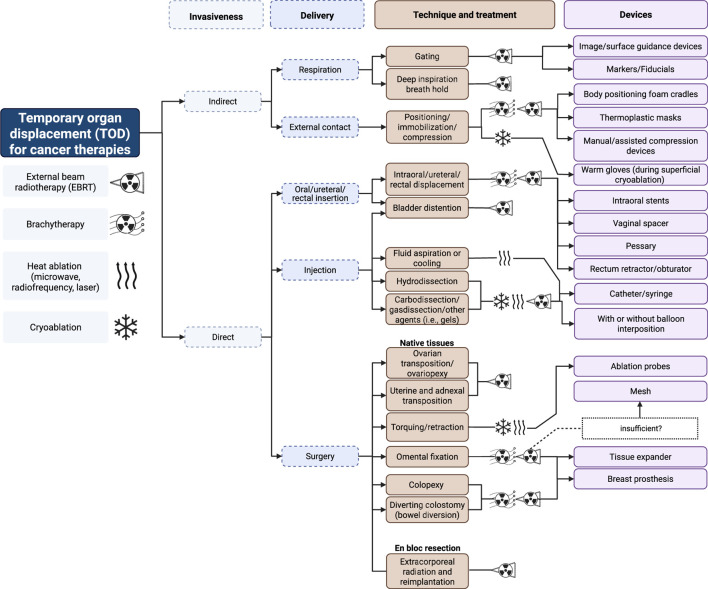
Classifications of TOD in cancer therapy. Created with BioRender.com.

We classify TODs primarily on invasiveness and mode of delivery followed by the specific methods and the necessary devices to achieve successful organ displacement. Figure 1 illustrates the nuances behind these classifications and show how they compare across different treatment modalities involving external beam RT (EBRT), BT, CA, or heat ablation (MWA, RFA, laser).

### 3.1 Indirect TOD

RT commonly employs indirect TOD techniques that makes use of physiological actions or external/physical aids to displace OARs. These techniques include deep inspiration breath hold (DIBH) and various immobilization methods, such as thermoplastic molds and specialized cradles. More specifically, this section explores two primary indirect TOD techniques for organ displacement: physiological manipulation and external contact.

#### 3.1.1 Physiological manipulation (swallowing and respiration)

**FIGURE 2:**
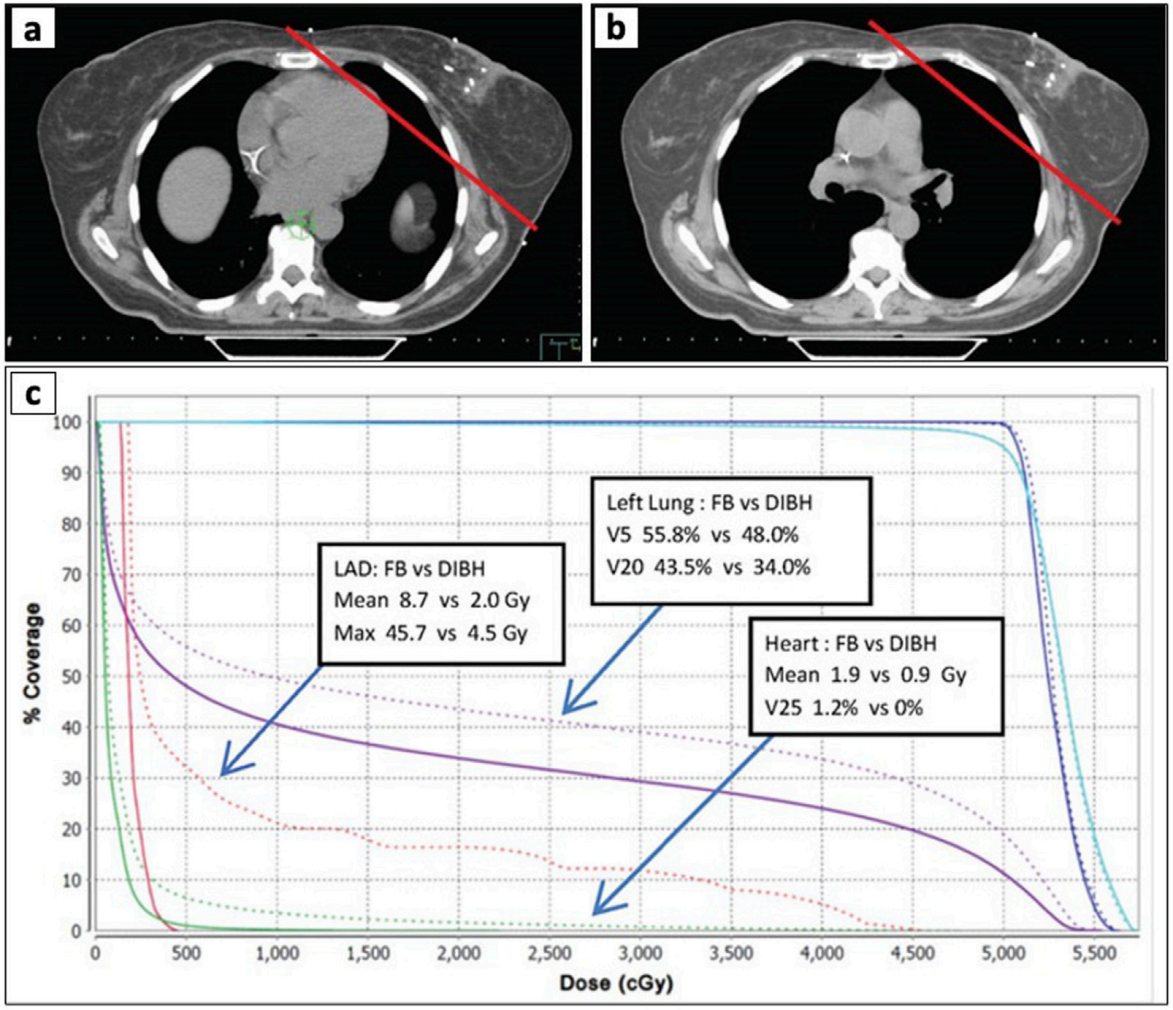
Comparison of Dose-Volume Histogram (DVH) during free breathing (FB) and deep inspiration breath hold (DIBH). **(a, b)** axial CT scan of the breast during FB (a) and DIBH **(b)**. The red line shows the radiation field path used for RT. **(c)** the DVH plot illustrates dose reductions in the left anterior descending artery (LAD), left lung, and heart during DIBH (solid lines) vs. FB (dotted lines).

Patients are often instructed to perform DIBH during RT to control their respiratory movements and lung-heart positions to minimize unwanted dose toxicities (see Figure 2) (60). Previous studies have demonstrated that DIBH can reduce non-target mean doses to the heart (15%–63%), medulla (15%–16%), and esophagus (6%–8%) without compromising target volumes during lung RT (61–64). Josipovic et al. (2019) ran a prospective trial assessing 69 patients with locally advanced non-small cell lung cancer (NSCLC) undergoing definitive RT, showing high compliance and target reproducibility in 50/69 patients (29). Another prospective trial also showed the efficacy of DIBH in patients with stage I/II breast cancer using an active breathing coordinator (ABC) device, showing a reduction in the affected heart volume during RT (with a mean absolute reduction of 1.5% in the heart expected tissue complication probability) (65). Similarly, the ABC device has been studied in patients with unresectable intrahepatic tumors undergoing RT, showing a reduction in normal liver irradiation while maximizing high-dose administration, and maintaining diaphragm and liver positions (66). While DIBH techniques and respiratory gating offer advantages in minimizing doses to OARs, it is important to note that the successful implementation of these methods depends on several factors guiding patient selection. These include the patient’s respiratory capabilities, the associated costs, convenience, and the potential benefits based on the tumor’s size, location, and type (67).

#### 3.1.2 External contact

**FIGURE 3:**
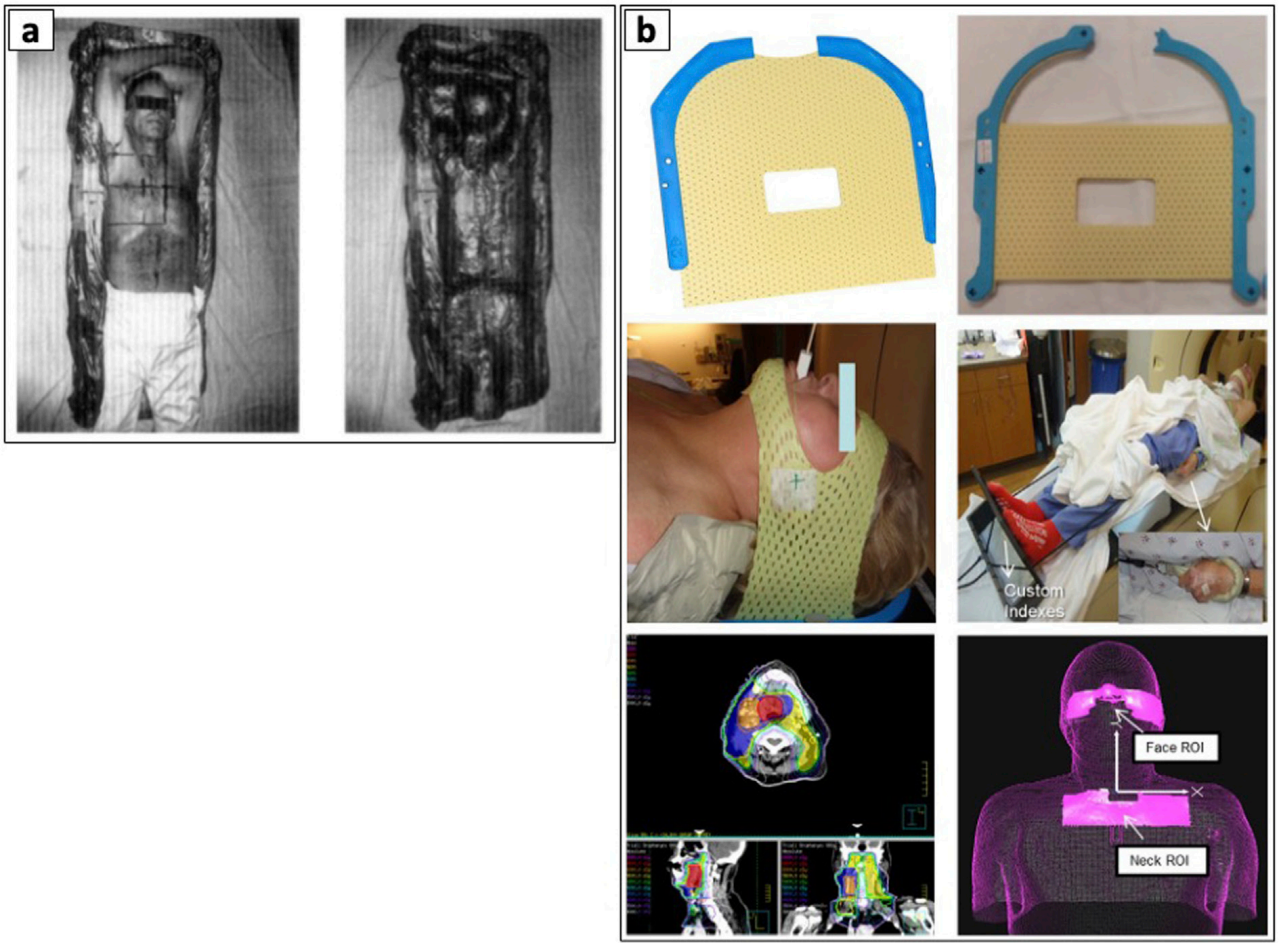
Examples of patient body immobilization devices. **(a)** Body foam. Reproduced with permission from “The prepared Styrofoam form (left), patient resting in the finished cradle (center), and the cradle without the patient demonstrating the close fit to the patient’s body (right)” by Gunilla C. Bentel, Lawrence B. Marks and Rupa Krishnamurthy licensed under CC-BY-NC-ND, and **(b)** Thermoplastic immobilization mask. Adapted with permission from “(a) Original short 3-point mask (Qfix, model RT-1876KSDGLF). (b) Mask modified with straight cuts at top and bottom. (c) Modified mask in place over only forehead and chin. (d) Overall patient setup. (e) Patient treatment plan with low neck coverage. (f) ROI selections on AlignRT relative to isocenter location. These two ROIs can be used to create a composite ROI for intrafractional tracking” by Bo Zhao, Genevieve Maquilan, Steve Jiang and David L. Schwartz, licensed under CC BY 4.0.

Prior literature demonstrated the feasibility and safety of using hand compressions and assisted compression devices to indirectly displace internal organ structures during before or during treatment. Here, Tuncali et al. (2006) demonstrated the effectiveness of manual hand compressions during magnetic resonance imaging (MRI) guided CA of renal tumors to displace the bowels (68). Other techniques showed the utility of external compression devices to achieve the optimal needle pathway and reduce the risk of injuring the bowel, bladder, and colon. Furthermore, these devices enable more accurate needle placement by providing stable compression and guidance, potentially increasing diagnostic yield and reducing the need for repeat biopsies (69, 70). More recently, 3D-printed compression paddles were developed to enhance needle access to difficult targets by allowing the displacement and immobilization of vital structures. These paddles safely guides the needles while ensuring the clinician’s hand is positioned away from the x-ray beam during computed tomography (CT) imaging to avoid unnecessary irradiation (71). In RT, non-invasive immobilization devices such as body foam cradles and thermoplastic masks have become essential components in treatment planning and delivery. For lung cancers, cervicothoracic immobilization devices have shown superior setup accuracy compared to traditional thoracoabdominal flat immobilization devices, particularly for patients requiring treatment of both primary lung lesions and supraclavicular lymph nodes (72). These devices significantly reduce organ motion and promote consistent patient positioning across multiple treatment sessions, with applications spanning various cancer types, including HNC (73–75), breast (76), lung (30, 31), prostate and bladder (77, 78), and paraspinal sarcomas (79) (see Figure 3).

### 3.2 Direct TOD

Direct TOD techniques involve actions involving the direct physical manipulation of OARs using spacers, to achieve targeted organ displacement with or without immobilization. These techniques are categorized based on their delivery method, which includes oral/ureteral/rectal insertion, injection, or surgical implantation. In this section, we discuss each delivery methods, the associated technique(s) and their unique advantages in managing organ positioning and immobilization during RT and locoregional ablation therapies.

#### 3.2.1 Oral, ureteral, and rectal insertion

##### 3.2.1.1 Intraoral stents

**FIGURE 4:**
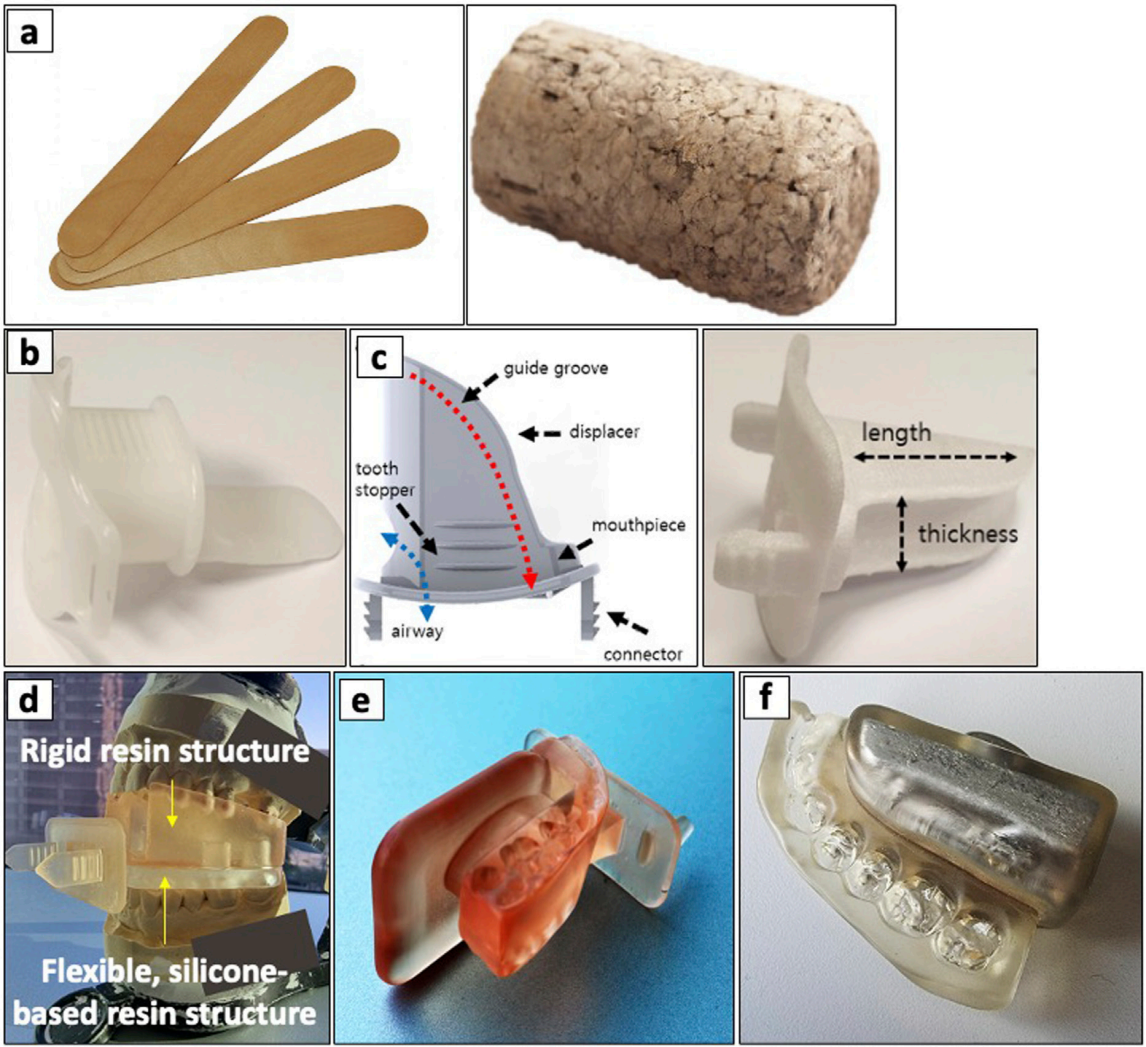
Types of intraoral stents ranging including non-customizable, semi-customizable, and customizable stents. **(a)** non-customizable stents including a stacked wooden tongue blade (left) and a cork (right). **(b)** a commercial tongue-depressing stent and **(c)** its semi-customized version (left) fabricated using 3D-printing (right). **(d)** a 3D-printed, customized tongue-depressing stent with flexible silicone-based resin structure, mounted on its dental impression. **(e)** a 3D-printed customized tongue-lateralizing intraoral stent. **(f)** a 3D-printed customized tongue-lateralizing stent filled with Lipowitz alloy. Adapted with permission from “a Top and b front views of the 3D model for the semi-customized tongue displacement device. It was printed using a 3D printer with a biocompatible material (c). d Commercially available standard mouthpiece, which has been the most commonly used device in H&N RT” by Chae-Seon Hong, Dongryul Oh, Sang Gyu Ju, Yong Chan Ahn, Cho Hee Na, Dong Yeol Kwon and Cheol Chong Kim, licensed under CC BY 4.0.

The rising incidence of tongue cancer in the 1980s and 1990s sparked interest in interstitial BT applications. However, its use resulted in adverse complications due to radiation-induced oral mucositis (RIOM). Radium needles and iridium-192 hairpins emerged as a simple yet effective approach to mitigating RIOM, enhanced by precise displacement and positioning of intraoral tongue stents (33–35, 80–82). In 1984, Niwa et al. demonstrated one of the earliest clinical uses of a tongue stent to separate the lower gingiva from the tumor and prevent osteoradionecrosis during interstitial BT (83). Subsequently, Fujita et al. (1993–1994) demonstrated that silicone intraoral stents provided superior dose reduction and shorter fabrication times than acrylic stents (84). They later demonstrated an improved dose-reduction effect when combined with a Lipowitz metal (85). Tamamoto et al. (1996) further advanced this field by developing mouth guard-like oral spacers using acrylic resin to accommodate dentate and edentulous patients (86). Further advancements in digital dentistry have allowed for customization, more efficient fabrication and improved patient comfort during and after treatment (36, 37, 87, 88). Traditional and modern approaches in the clinic utilize several types of intraoral stents, such as shielding prostheses (82, 89), radiation carriers, positioning stents (36, 37, 87, 88, 90–95), and radiation mouthguards (96). These stents differ in their specific treatments, advantages, disadvantages, and materials used in their creation, offering a range of options to suit individual patient needs and treatment requirements (see Figure 4) (82).

##### 3.2.1.2 Intravaginal/rectal spacers

Few studies have shown that silicone-based materials in the form of repurposed breast implants and tissue expanders can effectively reduce radiation exposure to surrounding tissues, making them valuable components in RT (97–99). For patients with stage Ib-IIb cervical carcinoma, receiving high-dose-rate BT (HDR-BT), the intravaginal insertion of silicone-based Foley balloons filled with contrast medium were demonstrated to reduce rectal and bladder doses (100, 101). Recently, Ates et al. (2022) evaluated silicone-filled vaginal spacers in patients with rhabdomyosarcoma, receiving proton beam RT (PBRT), demonstrating effective vaginal wall displacement and reduced radiation exposure (21). Other areas involving the use of ureteral and rectal stent spacers, along with bladder distention devices, have shown promise in protecting patients with abdominal and pelvic malignancies receiving RT. Here, self-expandable ureteral stents were able to reduce radiation doses to pelvic structures in patients with cervical or rectal cancer, with significant improvements in stent patency and postoperative complications (102). Others including rectal retractors offer a less invasive alternative to surgery and injection, resulting in more successful sparing with lower complication rates (15, 16, 103, 104).

#### 3.2.2 Injection-based TODs

Technological advancements in percutaneous abdominal ablation have led to the adoption of RFA and MWA for liver and kidney malignancies (105). While effective in achieving enhanced local tumor control, previous studies highlight potential risks to adjacent OARs, such as cholecystitis and gallbladder perforation (106). Liang et al. (2009) retrospectively analyzed 1,136 patients undergoing MWA for hepatocellular carcinoma (HCC), cholangiocarcinoma (CCA), and cholangio-HCC, reporting a 0.2% treatment-related mortality rate and a 2.6% major complication rate. Although complications were infrequent, their severity, ranging from liver abscess and bile duct injury to tumor seeding, hemorrhage, and skin burns, often resulted in significant morbidity and disability (107). These consequences have led to the development and adoption of injection techniques, such as hydrodissection, primarily used for lens-cortex separation and needle biopsy applications (108, 109). Other techniques involve the local administration of simple liquid solutions involving saline (110, 111) to more sophisticated hydrogel spacers (HS) (41, 112).

##### 3.2.2.1 Hydrodissection

**FIGURE 5:**
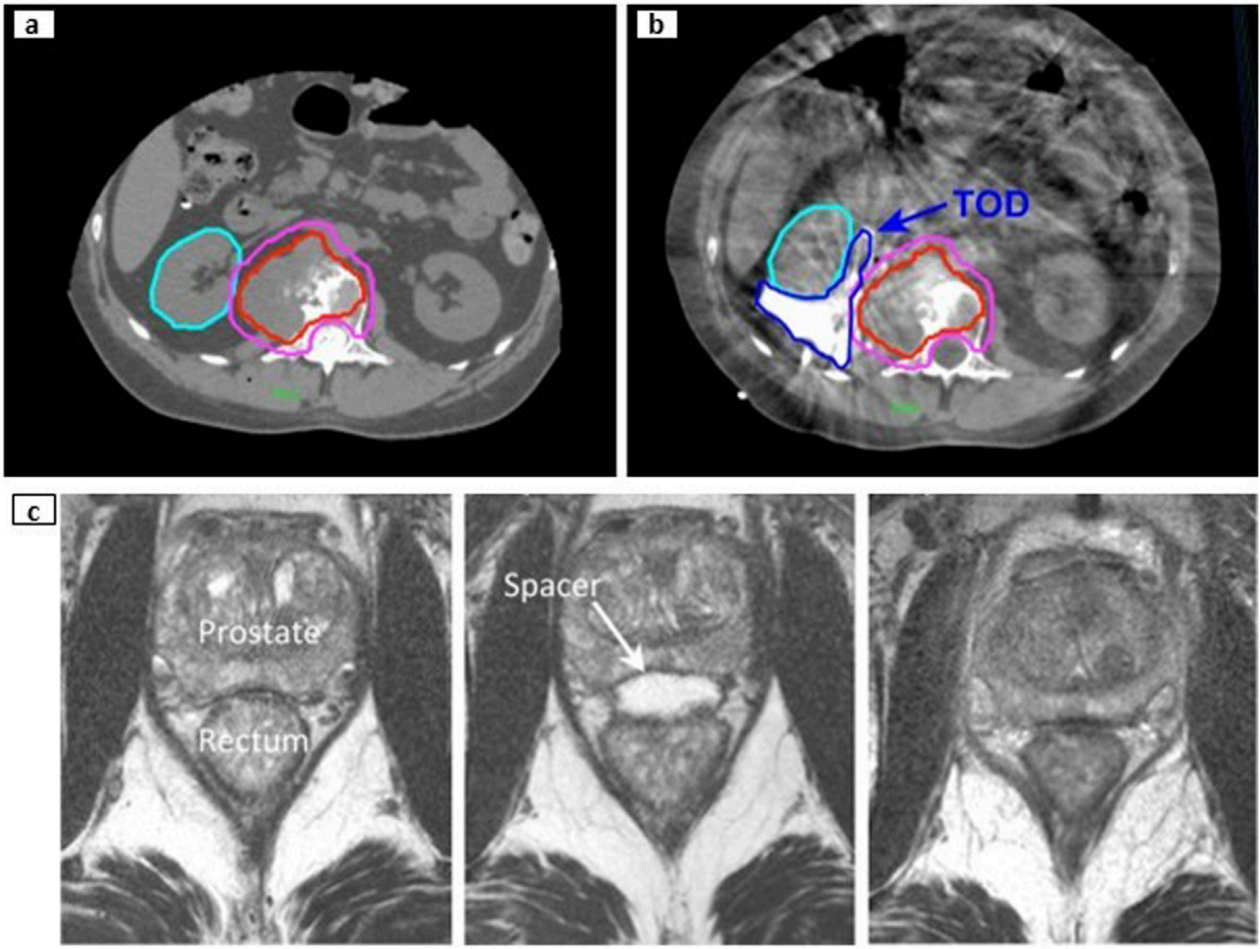
Examples of injectable spacers. **(a)** Baseline CT scan of a sacral chordoma, highlighting the proximity of the pelvic sacral tumor to the bowel at the S2-4 vertebrae. **(b)** Cone-beam CT image demonstrating successful temporary organ displacement (TOD) of the bowel, achieved through the injection of sterile saline via hydrodissection, creating a protective margin between the bowel and the tumor target. (a) and (b) Reproduced with permission from “Axial imaging of paraspinal non-seminomatous germ cell tumor treated to 2850 cGy” by Evangelia Katsoulakis, Stephen B Solomon, Majid Maybody, Douglas Housman, Greg Niyazov, Nadeem Riaz, Michael Lovelock, Daniel E Spratt, Joseph P Erinjeri, Raymond H Thornton and Yoshiya Yamada, licensed under CC BY 2.0. **(c)** Visualization of the TOD space between the prostate and rectum using SpaceOAR™ across varying timepoints. The SpacerOAR™ is a polyethylene glycol hydrogel material designed to spare the rectum during RT. Reproduced with permission from “T2-weighted magnetic resonance images of a spacer patient at baseline (a), post-application (b), and 12 months after spacer application (c)” by Mariados et al., licensed under CC-BY-NC-ND.

Hydrodissection utilizes fluid solutions to create a localized increase in pressure near the target organ, subsequently pushing OARs away from the treatment area. Standard hydrodissection procedures use local anesthetic, saline, 5% dextrose in water (D5W), or a dilute mixture of contrast agents for enhanced imaging visualization (see Figures 5a, b). Compared to other solutions, D5W is preferred due to its hypo-osmolar property, reducing risks of osmotic stress, and its slower absorption rate ideal for prolonged OAR separation. This is especially true for RFA procedures, where the use of D5W is the only fluid recommended due to its non-ionic composition, avoiding the spread of current and subsequent collateral damage to nearby tissues (113). Clinical studies have demonstrated the benefits of hydrodissection in various cancer treatments, showcasing its ability to protect OARs during biopsy procedures, RT, MWA, and RFA (109–111, 114–117). Studies have also demonstrated the safety and efficacy of hydrodissection in patients with papillary cancers receiving MWA and RFA (118–122). Furthermore, the infusion of saline into ureters, also known as ‘pyeloperfusion,’ has been shown to protect the urothelium, reducing the risks of urinary stricture and urinoma due to thermal damage from RWA, MWA, and CA (24). More recently, studies show that the injection of thermoprotective gels significantly reduce thermal damage in MWA, with similar benefits in image-guided biopsies, CA of the mediastinal, RFA of thyroids, and retroperitoneal tumor ablation (27, 118). As imaging techniques advanced, the need for visualization of the hydrodissection space developed, leading to using contrast agents. Using a 1:20 dilute mixture of iohexol-140 (Omnipaque; GE Healthcare, Princeton, New Jersey) in normal saline has been shown to improve the visibility of the fluid space under CT or MRI guidance, enabling controlled hydrodissection procedures (123). Advanced methods include the combination of balloon spacers with agents like collagen, hyaluronic acid (HA), saline with 5%–10% iohexol, fibrillar collagen, and thermoprotective gels (poloxamer 407) (124, 125). A multi-center study in prostate cancer confirmed that transperineal injection of a biodegradable balloon, ProSpace™ (BioProtect Ltd., Israel) between the prostate and rectum reduces rectal doses and improves immobilization during RT (126). Additionally, using multiple probes during MWA yields larger, symmetrical ablation zones and reduces treatment time (127). Andresciani et al. (2023) reported that dual-probe MWA with hydrodissection in 55 patients with HCC and unresectable liver metastases achieved safe ablation margins (2.5–166 cm^3^) for tumors (4.4–85 cm^3^) (128). Apart from liquid, air injection using a Chiba needle was employed to displace the small bowel during kidney RFA (2). However, this technique carries a higher risk of pain and potentially fatal air embolism if introduced intravenously. To address this limitation, Johnston et al. (2024) utilized a technique called ‘Carbodissection’, also known as ‘CO2 dissection’ or ‘gasdissection’, by injecting a highly soluble CO2 gas to displace the bowels during CA (56).

##### 3.2.2.2 Hydrogel spacers (HS)

Transperineal injections of HA reduced rectal toxicities in prostate cancer RT (130, 131), but HA’s rapid degradation under radiation and its high viscosity limited its effectiveness (132, 133). Concurrently, bioabsorbable HS made from polyethylene glycol (PEG) emerged as a more reliable alternative, including SpacerOAR™ (Augmenix Inc., Waltham, MA, US) and DuraSeal^®^ (Covidien, Mansfield, MA, US) (41, 134–137). Other bioresorbable hydrogel-based spacers made from hydroxypropyl methylcellulose (HPMC), such as Viscomet^®^ (Sun Pharmaceutical Industries Ltd., Mumbai, India), have also demonstrated feasibility and safety primarily in patients with cervical cancers receiving BT (138). Moreover, Weber et al. (2012) found that transperineal injection of HS reduced rectal doses and improved target coverage in more advanced techniques involving intensity-modulated RT (IMRT), volumetric-modulated arc therapy (VMAT), and intensity-modulated proton therapy (IMPT) (126).

Compared to SpaceOAR™ and DuraSeal^®^’s ‘passive’ reversibility through natural biodegradation, non-animal, stabilized HA (NASHA^®^) formulations such as Barrigel™ (Palette Life Sciences, now acquired by Teleflex Inc., US) exhibit ‘active’ reversibility characteristics. The reversible nature of Barrigel™ provides additional safety advantages, as demonstrated by Hong et al. (2022), where rectal wall infiltration was successfully reversed with hyaluronidase administration without adverse effects (140). A multi-center randomized controlled trial conducted across the US, Australia, and Spain have further shown the effectiveness of Barrigel™ in 136 hypofractionated prostate cancer patients, with improved spacing maintenance and safety profile (141). The study demonstrated that 98.5% of patients treated with Barrigel™ achieved ≥25% reduction in radiation dose to the rectum, with patients averaging an 85% dose reduction. Importantly, this translated to significantly reduced acute grade 2 GI toxicity (2.9% vs. 13.8% for control group, p = 0.01). PEG-based spacers have similarly established strong evidence, with SpaceOAR™ studies demonstrating efficacy in reducing rectal ulcer incidence and increasing prostate-rectum distance in SBRT patients receiving 45 Gy in 5 fractions (142). A phase III trial with 222 patients confirmed significant reductions in late rectal toxicity with no device-related complications (see Figure 5c) (129), with 3-year follow-up data highlighting sustained benefits and reduced medical treatment requirements for bowel complications (143–145). Recent research further showed SpaceOAR™'s particular effectiveness in patients with larger prostates, significantly reducing high-dose rectal exposure and GI toxicities (146).

Given these benefits, post-prostatectomy RT (PPRT) represents an expanding application area requiring specialized investigation, requiring a well-designed study with clear criteria to assess the clinical effectiveness of HS (147). Hong et al. (2024) conducted a retrospective study evaluating 64 patients who received PPRT, showing the effective and safe use of SpaceOAR™ in at least 95% of patients (148). Furthermore, the ongoing landmark trial, the Barrigel^®^ PPRT trial (NCT06496256, Teleflex Inc., US), aims to establish definitive efficacy in this patient population with the reduction in rectal volume receiving the prescribed dose as the primary endpoint. Beyond prostate applications, cervical cancer patients receiving HDR-BT were shown to benefit from other hydrogel-based spacers such as Suvenyl^®^ (discontinued, Chugai Pharmaceutical Co., Tokyo, Japan) (149–155), MucoUp^®^ (Seikagaku Co., Tokyo, Japan) (156, 157), and TraceIT^®^ (Augmenix Inc., US) (158). More specifically, a study by Iijima et al. (2021) using Suvenyl^®^ has shown that greater thickness and length of gel spacers lead to lower 2-cm^3^ covering doses to the rectum (152), though quality of evidence remains limited and requires further validation through larger prospective trials (42). In GI applications, Narang et al.’s (2024) study of six pancreatic adenocarcinoma patients injected with TraceIT^®^ successfully separated the pancreas head and duodenum with a mean spacing of 0.77 cm without any device-related adverse events and showed no damage to the duodenum in patients undergoing Whipple resection (159). Additionally, studies have shown promising outcomes of Barrigel™ in retroperitoneal settings, with a recent report by Lee et al. (2025) demonstrating successful large bowel displacement during SBRT for adrenal and renal malignancies (160).

Current literature demonstrates excellent patient tolerance of HS implantation overall. However, documented complications include rectal discomfort, pain, bacterial prostatitis, and perineal abscess, though these occur rarely (134). Technical limitations require consideration, as imaging artifacts serve as contraindications for HS use (161). The reversibility advantage of NASHA^®^ formulations over PEG-based alternatives provides enhanced safety profiles in cases of misplacement or complications, making stabilized HA an increasingly attractive option for complex anatomical applications where precision and safety are paramount.

#### 3.2.3 Surgically placed TODs

Advanced interventions become necessary when high radiation doses increase the risk of nearby organ damage (162). Techniques like ovarian transposition to preserve fertility during pelvic irradiation for Hodgkin’s disease (163), colpopexy adapted from colorectal surgery for TOD (164), and extracorporeal procedures requiring reimplantation of resected bone after RT (165). This section focuses on surgical techniques for TOD during RT and discusses specific aspects of implantation, probe manipulation for organ positioning and immobilization in locoregional ablation therapies.

##### 3.2.3.1 Surgery with implants

**FIGURE 6:**
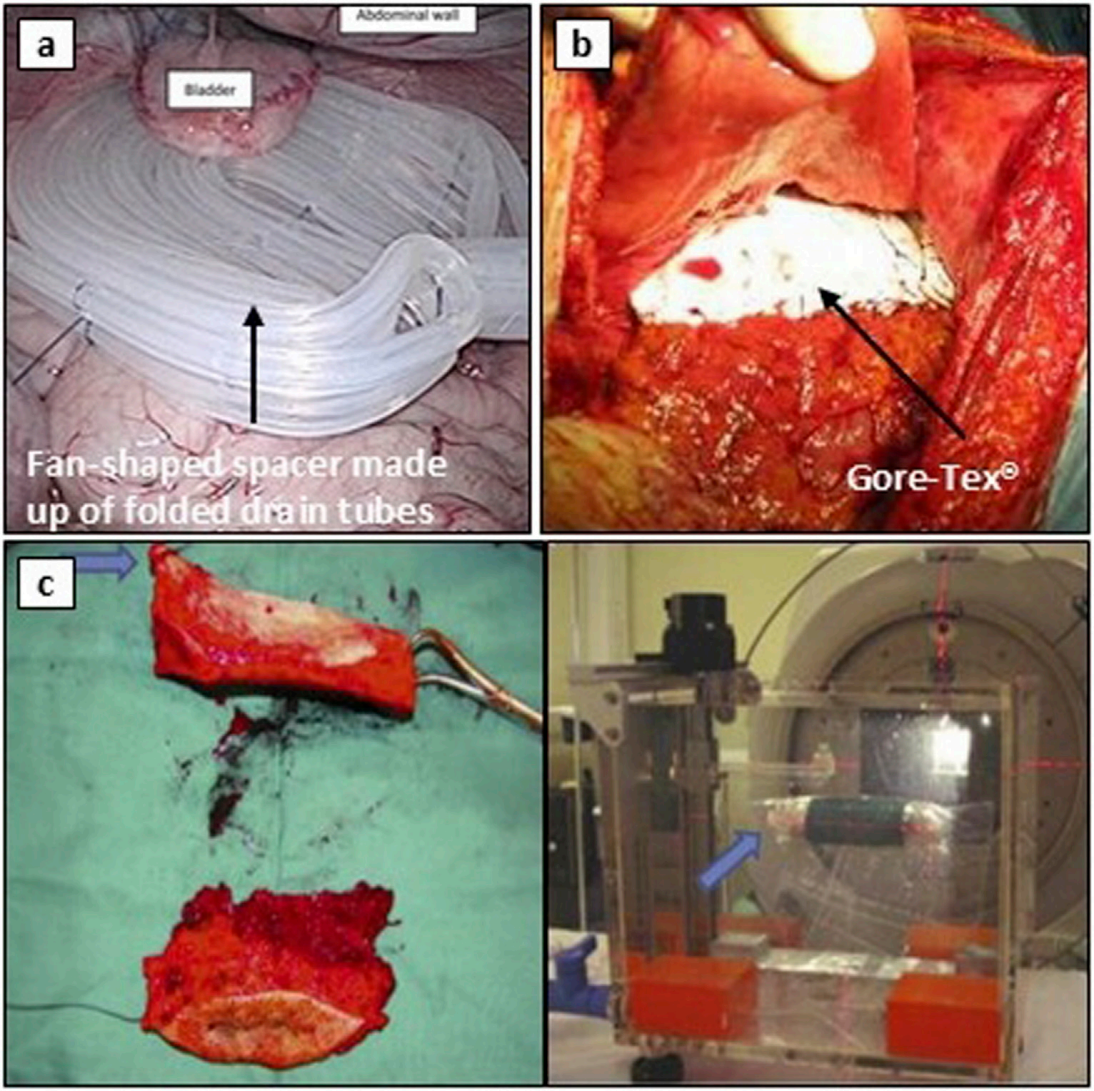
Examples of surgical TOD techniques. **(a)** a fan-shaped tube spacer inserted into the pelvic cavity of a porcine model, fixed to the abdominal walls using sutures. Adapted with permission from “Removal of the fan-shaped spacer in an animal model” by Norio Kubo, Takehiko Yokobori, Ryo Takahashi, Hiroomi Ogawa, Navchaa Gombodorj, Naoya Ohta, Tatsuya Ohno, Hiroshi Saeki, Ken Shirabe and Takayuki Asa, licensed under CC BY 4.0. **(b)** use of a Gore-Tex® Soft Tissue Patch between pancreas and GI tract. Adapted with permission from “Images of representative patients who underwent surgical spacer placement” by Dongha Lee, Shohei Komatsu, Kazuki Terashima, Hirochika Toyama, Yoshiro Matsuo, Daiki Takahashi, Masaki Suga, Naoko Nishimura, Kentaro Tai, Masahiro Kido, Yusuke Demizu, Sunao Tokumaru, Tomoaki Okimoto, Ryohei Sasaki and Takumi Fukumoto, licensed under CC BY 4.0. **(c)** setup of extracorporeal irradiation, with resected bone (left) wrapped for radiation (right). Reproduced with permission from “After resection of the tumor-bearing segment obvious tumor tissue as well as the biopsy tract are removed, whereas important functional structures such as the patellar tendon (⇨) are spared” by Andreas H. Krieg, Ulrich Lenze, Leandra Schultze, Markus W. Gross and Martin Haug, licensed under CC-BY-NC-ND.

Synthetic implants are essential in surgical TOD, as they support and displace OARs to minimize radiation exposure. This is particularly important for GI cancer patients who have undergone prior resections, such as abdominoperineal resection and pelvic exenteration, where cavities must be filled to protect surrounding organs during RT (166). Sugarbaker et al. (1983) introduced a plastic mesh to fill pelvic cavities in post-operative EBRT patients, reducing radiation enteritis from 40% to less than 5% (167). Later studies show the utility of the Dexon mesh to displace and immobilize the small bowel during RT, with no cases of radiation enteritis or mesh-related complications in a study of 60 patients, 92% of whom reported improved QOL (168). Polyglycolic acid (PGA) meshes, widely used for TOD, dissolve over time to avoid surgical removal (169). Building on this experience, Sezeur et al. (2008) combined polyglactine 910 (Vicryl) mesh with a saline-filled silicone balloon, achieving a 95% success rate in preventing small bowel irradiation in 50 patients (170). Similarly, Jesseph et al. (2013) used tissue expanders and breast prostheses to protect the small bowel during PBRT with no complications (171), and Chan et al. (2019) demonstrated the effectiveness of a tissue expander combined with an absorbable pelvic mesh sling for protecting the small bowel during pelvic RT (172). Interestingly, it has been shown that silicone film drains folded in a fan-shaped configuration successfully separated the small intestine from the pelvic space in a porcine model, and were subsequently removed without invasive surgery (173) (see Figure 6a). Another study used a silicone vaginal insert, known as the ‘Gelhorn pessary’, to manage pelvic organ prolapse during chemo RT of the cervix, a strategy that may help minimize unnecessary irradiation to surrounding healthy tissues (174).

Recent studies have demonstrated expanded polytetrafluoroethylene (ePTFE) Gore-Tex^®^ sheets, a biocompatible, flexible, and impermeable material, for cosmetic means and TOD procedures (175). Gore-Tex^®^ spacers can protect the GI tract during PBRT for locally advanced pancreatic cancer with improved tumor volume coverage while adhering to GI dose constraints (22) (see Figure 6b). Additionally, researchers have applied Gore-Tex^®^ in image-guided IMRT for paraspinal tumors, PBRT for unresectable HCC, and other abdominal, pelvic, and retroperitoneal malignancies (176–178). More advanced materials, such as biologic devices made from natural tissues or biologically derived materials, offer superior biocompatibility compared to synthetic alternatives. A 2014 trial demonstrated that biologic meshes are more resistant to infection, resulting in fewer hernias and reinterventions (179). A biologic mesh derived from sterile cadaver skin, AlloDerm™ (LifeCell Corporation, acquired by Allergan Aesthetics, US) has been used as an implantable spacer for liver and pelvic tumors treated with RT (180, 181). In addition, Alloderm™ has also been demonstrated to displace OARs effectively, such as the small bowel, colon, and pancreas during PBRT and IMRT, minimizing GI toxicities (23, 182, 183).

##### 3.2.3.2 Surgery with native tissues

While invasive, using the patient’s native tissues for surgical TOD techniques offers the most biocompatible method for displacing and securing organs, minimizing complications, and protecting healthy tissues during RT. Colopexy, a procedure that fixates the colon to the abdominal wall, was traditionally used for recurrent volvulus but has been adapted for TOD. The technique now includes using an omental ‘flap’, ‘sling’, or ‘envelope’ as a spacer to prevent dose toxicities to the bowels (184–186). Russ et al. (1984) demonstrated that omental transposition flaps achieved a 95% success rate in bowel displacement, reducing radiation enteritis from 30% to under 5% and improving QOL for 90% of patients (187). Another study demonstrated the feasibility of suturing the bladder to the abdominal wall and retroperitoneum, then distended with fluid to shield other peritoneal contents from radiation (52). Similarly, suturing the greater omentum to displace and immobilize the small intestines can effectively move them away from radiation targets (53). Uterine and adnexal transposition is a surgical technique used to protect reproductive organs from radiation damage, preserving fertility in women undergoing pelvic RT (189, 190). Ovarian transposition, also known as ovariopexy, has been used since the 1980s to protect the ovaries and fallopian tubes from radiation, reducing infertility during high-dose pelvic radiation for cancers such as Hodgkin’s disease, cervical and rectal cancers (191–193). More recently, Ribeiro et al. (2024) conducted a longitudinal study from 2017 to 2024 on laparoscopic transposition of the uterus and adnexa to the upper abdomen during pelvic RT, restoring them via rectosigmoidectomy post-RT. In a study of eight patients, six maintained uterine preservation with no significant complications. Follow-ups showed cervical ischemia in three patients, one death from carcinomatosis, and two successful pregnancies (194, 195).

##### 3.2.3.3 Extracorporeal radiation and reimplantation

Extracorporeal irradiation and reimplantation is a surgical procedure for TOD in which surgeons remove a tumor-bearing bone segment, irradiate it with high-dose radiation outside the body, and then reimplant it free of tumor cells (196–198). First reported by Spira and Lubin in 1968, the technique aimed to preserve limb function and prevent amputation (165). A study by Davidson et al. (2005) involved 50 patients with bone malignancies who received 50 Gy of extracorporeal irradiation. At a mean follow-up of 38 months, 42 out of 50 patients were alive and disease-free. Functional outcomes were favorable, with a mean Musculoskeletal Tumor Society score of 77 and a Toronto Extremity Salvage score of 81 (199). A recent study by Krieg et al. (2019) on wide resection and reimplantation of irradiated autografts in patients with sarcomas showed good to excellent functional outcomes in 7 of 8 patients, demonstrating the potential of this technique for limb salvage in cases of tibial sarcomas (200) (see Figure 6c).

##### 3.2.3.4 Probe manipulation

A simple approach for minimizing locoregional ablation damage to OARs involves probe manipulation, such as elevation or retraction. In CA procedures, clinicians carefully adjust a cryoprobe to target the tissue while avoiding adjacent structures (58). In renal tumors involving the chest wall, elevating the probe increases separation from the chest wall and pectoralis muscle while retracting protects the bowel. Aside from elevation and retraction, a similar probe manipulation technique involves applying torque or a rotational force to the ablation probe. Probe torquing can also temporarily displace organs to create space, as observed in cases of kidney tumors. Here, torquing the probe can shift the kidney position and increase the distance from the nearby bowel, preventing thermal injury to OARs during the subsequent freezing cycle. Adding gentle back-and-forth motion of the cryoprobe during the procedure prevents the probe from adhering to frozen tissue and allows minor adjustments in probe position to optimize ice ball formation and coverage (58, 201, 202). Clinicians often combine probe manipulation techniques with injections and surgical implantations. Through this, interventional radiologists can further reduce the risk of collateral damage during CA procedures near sensitive OARs (2, 55).

## 4 Spacers in the clinic

**FIGURE 7:**
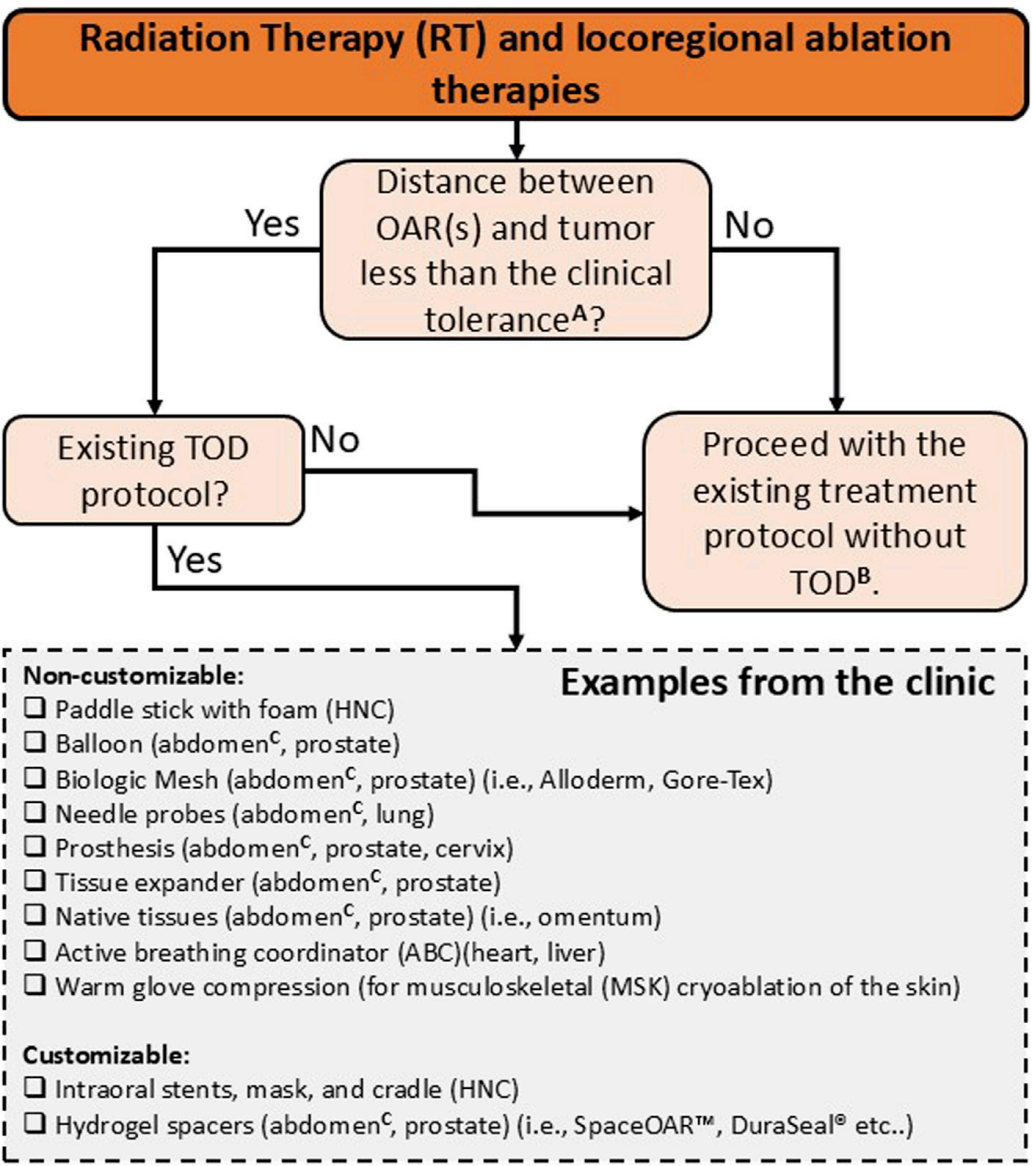
Indications and contra-indications for TOD use in RT and locoregional ablation therapies. Non-customizable devices, such as standard balloons and generic spacers, are designed for general use without the need for patient specificity. Customizable devices, including patient-specific spacers and immobilization systems, are tailored to fit unique anatomical features, potentially improving precision and comfort. ^A^ this clinical threshold is not universally defined and varies by modality, organ site, and image guidance capabilities. In RT, particularly with advanced modalities such as VMAT and IGRT, sub-centimeter threshold (<1 cm) between tumor and OARs are often feasible, but larger separations (>1 cm) may also require spacer use depending on individual anatomy, organ motion, and setup uncertainties. For percutaneous ablation, most studies use a <2 cm threshold to ensure complete tumor coverage and adequate OAR protection, but actual clinical tolerance should be tailored to patient-specific anatomy and organ-specific ablation protocols. ^B^ target structures are often underdosed to prioritize dose constraints to OARs in RT. In percutaneous ablation cases, target structures are treated at reduced power (watts) and ablation time to minimize collateral damage to adjacent healthy tissues. ^C^ abdomen includes liver, kidney, pancreas, and bowels.

This section reviews key clinical factors and their role in promoting a safe and effective TOD. These include accurate patient selection, organ separation, immobilization, resource availability, spacer design, placement, and positioning verification. Figure 7 illustrates an overview of the current clinical landscape of TOD techniques in RT and locoregional ablation therapies. Although numerous studies have examined TOD, consensus regarding the indications and contraindications for TOD techniques remains unclear and current recommendations are based solely on the clinical factors outlined below.

### 4.1 Organ separation and immobilization

Intuitively, the tumor’s location and its distance from OARs provide crucial information for determining whether TOD is necessary and, if so, which specific TOD technique would be most appropriate. Tumor proximity to OARs indicates the need for intraoral stents, organ delineation, DIBH, or immobilization techniques in RT and hydrodissection or probe manipulation in locoregional ablation therapies. A standard method to measure these distances involves the manual measurement and annotation of CT slices, measuring the distance between boundary edges of 2D contours. More efficient and automated measurement methods utilize existing Euclidean pairwise distance or K-nearest neighbors (KNN) algorithms to measure the closest surface distance between 3D volumetric contours.

For RFA and MWA, tumors within 1 cm separation distance to OARs, such as the GI tract, diaphragm, gallbladder, major blood vessels, or bile ducts, indicate the necessity for hydrodissection (25, 203). Numerous studies have shown that maintaining a separation of at least 1 cm between the treatment margin and OAR(s) minimizes thermal damage (204–208), with hydrodissection achieving >1.5 cm separations (209). Ginat et al. (2010) recommend a safety margin of at least 2 cm for adjacent bowels to avoid complications (55). Although proximity to OARs presents challenges, successful ablations have been performed within 1 cm of these structures. Some patients achieved safe distances of 0.5–0.9 cm with hydrodissection (25, 119, 121, 203, 205). Moreover, researchers have demonstrated that minimally invasive manual hand compressions achieve a mean separation distance of 2.6 cm between the bowels and the renal tumor during CA (68).

RT centers use comparable tolerances, particularly for oropharyngeal, GI, and GU cancers. In HNC, past literature suggests intraoral stent thickness of >1 cm to prevent osteoradionecrosis during interstitial BT (84). Barcellini et al. (2022) highlighted the opportunity for omental flap spacers in patients with pelvic malignancies for carbon ion RT when tumor to OAR distance falls below 0.5 cm (184). In prostate cancers, HS injection has become increasingly prevalent, effectively reducing rectal toxicity by achieving >1 cm away from the prostate (129, 143, 210, 211). GI cancers have benefited from applying biologic mesh spacers, achieving immobilization of OARs and separation distances ranging from 0.5 to 4 cm in patients receiving PBRT, IMRT, and SBRT (23, 110). Tables 1, 2 present prospective clinical studies evaluating spacer techniques in patients receiving RT and locoregional ablation therapies, respectively. Studies were selected based on reporting both clinical treatment outcomes and quantitative measurements of achieved organ separation distances following spacer implementation.

**TABLE 1:** Prospective clinical studies primarily reporting post-spacer distance measurements from patients (pts) receiving RT with TOD spacers.

Study type	No. of pts	Age	Cancer type(s)/site(s)	Treatment modality	OARs	TOD technique(s)	TOD spacer(s)	Pre-spacer distance (cm)	Post-spacer distance (cm)	Reported complication(s) related to spacer	Ref.
Prospective, single-arm clinical trial	27	median of 67 (range: 55–77)	prostate cancer	Mono-BT (HDR/LDR), IMBT (HDR), EBRT	rectum	transperineal injection of HA in the perirectal fat guided by TRUS	3–7 mL HA	not reported	mean of 2 cm	none directly related to spacer use	Prada et al. (2007) (124)
Case report	14	mean of 38 (range: 30–43)	cervical and vaginal cancers	EBRT	ovaries	Bilateral ovarian transposition	n/a	not reported	range: 3–4 cm above umbilical line	none directly related to spacer use	Huang et al. (2007) (213)
Prospective comparative cohort study	32	mean of 68, median of 69 (range: 55–78)	prostate cancer	LDR-BT	rectum	transperineal injection of HA in the perirectal fat guided by TRUS	6–8 mL HA	not reported	≥2 cm	2 pts experienced edematous and congested mucosa	Prada et al. (2009) (214)
Prospective cohort study with a historical control	10	median of 62 (range: 66–88)	prostate cancer	HDR-BT, IMRT	rectum	transperineal injection of cross-linked hyaluronan gel/HA guided with TRUS	9 mL Hylaform Genzyme Corporation, Cambridge, MA)	not reported	range: 0.8–1.8 cm	none directly related to spacer use	Wilder et al. (2010) (215)
Prospective cohort study	18	mean of 71	prostate cancer	3DCRT, IMRT	rectum	transperineal hydrogel injection guided by TRUS	10 mL SpaceOAR™	range: 0.1–0.5 cm	range: 0.9–1.1 cm	none directly related to spacer use	Pinkawa et al. (2011) (210)
Prospective cohort study with control group	30	median 70	prostate cancer	HDR-BT, IMRT	rectum	transperineal injection of cross-linked hyaluronan gel/HA guided with TRUS	9 mL Hylaform Genzyme Corporation, Cambridge, MA)	not reported	median of 1.3 cm (range: 0.6–1.9 cm)	none directly related to spacer use	Wilder et al. (2011) (216)
Prospective case series, non-randomized, multi-center, single-arm, open-label	29	mean of 67	prostate cancer	IMRT	rectum	transperineal hydrogel injection guided by TRUS	10 mL SpaceOAR™	mean of 0.48 ± 0.24 cm	mean of 1.47 ± 0.52 cm	none directly related to spacer use	Hatiboglu et al. (2012) (217)
Retrospective cohort study	53	range: 22 to 48	cervical cancer	HDR-BT, EBRT	ovaries	lateral ovarian transposition to the paracolic gutters with or without radical hysterectomy and lymph node dissection	n/a	not reported	>1.5 cm above illiac crest	none directly related to spacer use	Hwang et al. (2012) (218)
Pilot study	11	not reported	prostate cancer	IMRT	rectum	transperineal injection of human collaged in the perirectal space guided by US	20 mL Cymetra (Allergan Aesthetics, US)	not reported	mean of 1.27 cm (range: 0.8–1.9 cm)	1 pt experienced urinary retention	Noyes et al. (2012) (131)
Prospective, multi-center, observational study	27	not reported	prostate cancer	IMRT, 3DCRT	rectum	transperineal implantation of a biodegradable balloon in the Denonvillier’s fascia, guided by TRUS	ProSpace™ balloon filled with saline (BioProtect Ltd., Israel) (length: 0.22–0.47 cm, height: 1.67–1.86 cm)	not reported	mean of 2.47 ± 0.47 cm	none directly related to spacer use	Gez et al. (2013) (211)
Retrospective case series	5	not reported	sarcoma (retroperitoneal and pelvic)	IMRT, PBRT, IORT	small bowel, colon, ureter, bladder, pancreas	surgical implantation of BMS	8 × 16 cm AlloDerm™ sheet with 0.23–0.33 cm thickness	mean of 0.13–0.9 cm across all adjacent OARs	mean of 0.8–2.35 cm across all adjacent OARs	none directly related to spacer use	Yoon et al. (2013) (183)
Prospective cohort study	5	not reported	prostate cancer	BT	rectum	transperineal hydrogel injection into the anterior perirectal fat under general anesthetic guided by TRUS	≈15 mL SpaceOAR™	mean of 0.26 ± 0.45 cm	mean 1.5 ± 0.34 cm	none directly related to spacer use	Beydoun et al. (2013) (220)
Case report	1	57	prostate cancer	ADT, HDR-BT	rectum	transperineal hydrogel injection guided by TRUS	10 mL SpaceOAR™	not reported	range: 1.4–1.5 cm	the pt experienced mild rectal bleeding (9 months post implantation)	Nguyen et al. (2013) (221)
Prospective case report	11	mean of 56 (range: 20–80)	chordoma, sarcoma, RCC, NSGCT (Non-seminomatous germ cell tumors)	IG-IMRT	bowels, kidney	hydrodissection	normal saline solution with 5%–10% iohexol	not reported	mean of 1.75 cm (range: 0.7–3.2 cm)	none directly related to spacer use	Katsoulakis et al. (2013) (111)
Prospective, multi-center, non-randomized, single-arm study	48	mean of 69	prostate cancer	IMRT	rectum	transperineal injection of hydrogel	10–15 mL SpaceOAR™	not reported	≈1 cm	1 pt experienced grade 1 proctitis	Uhl et al. (2013) (222), Song et al. (2013) (223)
Case report	3	not reported	rectal and cervical cancers	BT	small bowel, colon	transperineal injection of hydrogel guided by TRUS	DuraSeal^®^	not reported	>1 cm	not reported	Viswanathan et al. (2013) (136)
Retrospective cohort study	11	median of 69 (range: 61–81)	prostate and rectal cancers	LDR-BT	rectum	transperineal injection of hydrogel guided by TRUS	5 mL DuraSeal^®^ diluted 1:1 with 5 mL saline	not reported	range: 0.37–1.55 cm	none directly related to spacer use	Mahal et al. (2014) (224)
Prospective cohort study	100	median of 66 (range: 48–84)	prostate cancer	HDR-BT, IMRT	rectum	transperineal injection of hydrogel in the anterior perirectal fat guided by TRUS	5–10 mL DuraSeal^®^	mean of 0.4 ± 0.2 cm	mean of 1.2 ± 0.4 cm	2 pts developed bacterial prostatitis, 1 pt developed bacterial epididymitis	Strom et al. (2014) (225)
Prospective cohort study	10	not reported	prostate cancer	LDR-BT	rectum	transperineal injection of hydrogel guided by TRUS	10 mL DuraSeal®diluted with saline	not reported	≈1 cm	1 pt reported a sensation of rectal pressure at follow-up, 1 pt experienced need for defecation	Heikkilä et al. (2014) (226)
Case report	1	66	prostate cancer	LDR-BT	rectum	transperineal injection of hydrogel guided by TRUS	≈10 mL SpaceOAR™	not reported	range: 0.6–1.5 cm	submucosal induration in the anterior rectal wall, possible mechanical or ischemic injury	Teh et al. (2014) (227)
Case report	51	mean of 49 (range: 28–71)	cervical cancer	EBRT, BT	small bowel	omental flap surgery	omentum	not reported	>1 cm from the posterior aspect of the cervical tumor	none directly related to spacer use	Leblanc et al. (2014) (185)
Prospective, multi-center, randomized controlled study	149	mean of 66	prostate cancer	IG-IMRT	rectum	transperineal injection of hydrogel	SpaceOAR™	mean of 0.16 ± 0.22 cm	mean of 1.3 ± 0.4 cm	none directly related to spacer use	Mariados et al. (2015) (129)
Case report	1	77	prostate cancer	IMRT	rectum	transperineal hydrogel injection guided by TRUS	10 mL SpaceOAR™	not reported	>1 cm	increased frequency of bowel movements and rectal discomfort	Pinkawa et al. (2015) (228)
Case report	1	56	cervical cancer	BT	colon and rectum	transperineal hydrogel injection through the recto-vaginal septum guided by TRUS	≈50 mL DuraSeal^®^	mean of 0.18 cm	mean width of 1.1 cm and length of 5 cm (between anterior rectal wall and the posterior vaginal wall)	none directly related to spacer use	Basu et al. (2016) (138)
Prospective cohort study	326	median of 74 (range: 46–96)	prostate cancer	HDR-BT, IMRT	rectum	transperineal injection of hydrogel in the anterior perirectal space guided by US	10 mL DuraSeal^®^ diluted with saline	not reported	≈1.6 cm	none directly related to spacer use	Yeh et al. (2016) (229)
Retrospective case report	7	mean of 50 (range: 30–62)	CCA, CRLM	IMRT, SBRT	stomach, duodenum, colon	surgical implantation of a BMS, secured to adjacent soft tissue or liver using 1 cm clips/intracorporeal sutures	20 × 16 cm AlloDerm™ sheet	not reported	mean of 2.1 cm (range: 0.37–4.16)	1 pt developed a superficial surgical site infection, 1 pt had postoperative ileus	Ismael et al. (2016) (182)
Prospective cohort study	74	median of 69	prostate cancer	LDR-BT, EBRT	rectum	transperineal injection of hydrogel in the Denonvillier’s fascia guided by TRUS	SpaceOAR™	not reported	median of 1.12 cm (range of 0.16–1.67 cm)	none directly related to spacer use (some pts reported sensation of fullness in the rectum)	Taggar et al. (2018) (230)
Case report	1	61	prostate cancer	HDR-BT, IMRT	rectum	transperineal injection of hydrogel in the Denonvillier’s fascia guided by TRUS	SpaceOAR™	range: 0.47–1.31 cm	range: 1.24–1.64 cm	none directly related to spacer use	Hepp et al. (2018) (231)
Prospective, phase II study	31	mean of 74 (range: 61–84)	pancreatic cancer	IMRT	rectum	transperineal injection of hydrogel in the Denonvillier’s fascia guided by TRUS	SpaceOAR™	not reported	mean of 1.5 cm, median of 1 cm (range: 0.5–2 cm)	none directly related to spacer use	Chao et al. (2019) (232)
Retrospective cohort study	150	not reported	cervical cancer	3DCRT, IMRT	ovaries	unilateral or bilateral ovarian transposition at the peritoneum of the paracolic sulci	n/a	not reported	>1.12 cm above the iliac crest	none directly related to spacer use	Lv et al. (2019) (233)
Case report	1	67	sacral chordoma	IMRT	small bowel	surgical implantation of a bioabsorbable mesh sling to compartmentalize the abdomen and pelvis a tissue expander and normal saline to support the sling and fill up the space within the pelvis	20 × 30 cm Vicryl mesh Bag (Ethicon, United States) 13.7 × 7.1 × 7.7 cm rectangular tissue expander (Mentor, United States) followed by 450 mL normal saline	not reported	>8 cm	none directly related to spacer use	Chan et al. (2019) (172)
Retrospective cohort study	31	mean of 59 (range: 20–80)	chordoma, sarcoma, RCC, GCT, ovary	SBRT	small bowel, left kidney, descending colon	hydrodissection	1:20 dilute mixture of iohexol-140 in normal saline (Omnipaque; GE Healthcare, Princeton, NJ)	median of 0.41 cm (range: 0.05–0.87 cm)	median of 2 cm (range: 0.67–3.93 cm)	none directly related to spacer use	Maybody et al. (2020) (110)
Prospective, single‐arm Phase II clinical trial	81	median of 68 (range: 52–79)	prostate cancer	3DCRT, IMRT	rectum	transperineal injection of HA guided with TRUS	15 mL Macrolane VRF 30 (Q-Med/Galderma, Uppsala, Sweden)	not reported	>1 cm from the Denonvillier’s fascia on the prostate	none directly related to spacer use (7 pts reported feeling of anal fullness a week after HA injection)	Björeland et al. (2023) (130)
Pilot study	5	median of 75 (range 66–79)	cervical cancer	BT	rectum, bladder	injection of hydrogel, guided with TRUS	5–10 mL to the bladder wall and 14–30 mL MucoUp^®^ (combined with 3 mL of contrast agent)	not reported	0.5–1.0 cm (anterior vaginal wall to the bladder wall) and 0.5–1.9 cm (posterior vaginal wall and rectal wall)	none directly related to spacer use	Muramoto et al (2023) (156)
Prospective, multi-center, randomized, single arm study	136	mean of 69 (range: 45–82)	prostate cancer	hypofractionated IG-IMRT	rectum	transperineal injection of NASHA^®^ guided with TRUS	9–12 mL of Barrigel™	not reported	1.29 ± 0.35 between the Denonvillier’s fascia and the anterior rectal wall	none directly related to spacer use	Mariados et al (2023) (141)
Prospective, multi-center, non-randomized, single arm study	6	median of 70 (range: 60–80)	pancreatic cancer	IMRT, SBRT	duodenum (PD groove)	injection of a radiopaque hydrogel guided by endoscopic US	TraceIT^®^ (90% water, 9.25% polyethylene glycol, and 0.75% iodine)	not reported	mean of 0.77 ± 0.24 cm	none directly related to spacer use	Narang et al. (2024) (159)

Abbreviations: Brachytherapy (BT); high-dose rate (HDR); low-dose rate (LDR); intensity-modulated BT (IMBT); external beam RT (EBRT); hyaluronic acid (HA); non-animal, stabilized HA (NASHA^®^); transrectal ultrasound (TRUS); ultrasound (US); intensity-modulated RT (IMRT); 3D conformal RT (3DCRT); proton beam RT (PBRT); intraoperative RT (IORT); biologic mesh spacer (BMS); androgen deprivation therapy (ADT); image-guided, intensity-modulated RT (IG-IMRT); renal cell carcinoma (RCC); non-seminomatous germ cell tumors (NSGCT); cholangiocarcinoma (CCA); stereotactic body RT (SBRT); giant cell tumor (GCT); pancreaticoduodenal (PD).

**TABLE 2:** Prospective clinical studies primarily reporting post-spacer distance measurements from patients receiving locoregional ablation therapies with TOD spacers.

Study type	No. of pts	Age	Cancer type(s)/site(s)	Treatment modality	OAR(s)	TOD technique(s)	TOD spacer(s)	pre-spacer distance (cm)	post-spacer distance (cm)	Reported complication(s) related to spacer	Ref.
Retrospective observational study	31	mean of 68 (range: 22–91)	RCC	RFA	colon, stomach, duodenum, small bowel, pancreas, left adrenal gland, left ureter, spleen, liver	hydrodissection	60 mL 5% dextrose in water (D5W)	mean of 0.36 cm (range: 0.1–1 cm) median of 0.3 cm mode of 0.3 cm	mean of 1.94 cm (range: 1.1–4.3 cm) median of 1.9 cm mode of 1.9 cm	None directly related to spacer use	Arellano et al. (2012) (209)
Retrospective cohort study	6	mean of 65 (range: 49–73)	HCC, CRC, breast cancer, TCC	RFA	gallbladder	bile aspiration from the gallbladder followed by hydrodissection	10–35 mL of 5% dextrose, 5% dextran, or 0.9% saline mixed with contrast 20–22G fine needle aspiration from the gallbladder	<0.4 cm	>0.5 cm	1 patient reported right sided pleural effusion, 1 patient reported hematoma in the gallbladder bed, and 1 patient reported hemorrhage in the area of ablation	Levit et al. (2012) (25)
Case report	2	45 and 49	RCC	RFA	bowels	hydrodissection, paranephric water instillation with real-time sonographic guidance	135–150 mL sterile water	not reported	range: 2.1–2.5 cm	None directly related to spacer use	Farrell et al. (2012) (59)
Case report	3	60 and 80	RCC	RFA	small bowel	air injection probe torquing/retraction	3 mL air injection using a Chiba needle probe manipulation using 2 cm probe	not reported	≈1 cm	None directly related to spacer use	Lidell et al. (2012) (2)
Retrospective cohort study	60	mean of 59	HCC, CRLM	MWA	portal/hepatic veins, GI tract, gallbladder, parenchyma organs	hydrodissection	0.9% normal saline with a 2% iodine contrast solution	mean of 0.213 ± 0.1 cm (range: 0–0.48 cm)	mean of 0.72 ± 0.13 cm (range: 0.6–1 cm)	None directly related to spacer use	Liu et al. (2021) (203)
Prospective cohort study	166	mean of 42	thyroid cancer	RFA	common carotid artery, trachea/esophagus, parathyroid, recurrent laryngeal nerves	hydrodissection	mean of 113.68 mL (range: 10–450 mL) 5% glucose	not reported	range: 0.3–0.5 cm	None directly related to spacer use (1 patient experienced hoarseness, 2 patients had hematomas, and 3 patients vomited)	Ma et al. (2021) (115)
Retrospective cohort study	341	median of 41 (range: 20–80)	thyroid cancer	MWA	trachea, esophagus, nerves, great blood vessels	hydrodissection	normal saline with 0.5% lidocaine mixture injected along thyroid capsule to relieve pain during ablation	not reported	>0.5 cm	Hoarseness caused by recurrent laryngeal nerve injury	Zhao et al. (2023) (119)
Retrospective cohort study	66	mean of 58	HCC	MWA	diaphragm, GI tract	hydrodissection	0.9% normal saline	not reported	>0.5 cm	None directly related to spacer use (postoperative complications in three patients: liver abscess and biliary injury)	Song et al. (2024) (121)

Abbreviations: temporary organ displacement (TOD); transitional cell carcinoma (TCC); microwave ablation (MWA); radiofrequency ablation (RFA); organs-at-risk (OARs); renal cell carcinoma (RCC); hepatocellular carcinoma (HCC); colorectal carcinoma (CRC); colorectal liver metastases (CRLM).

In conjunction with organ displacement, clinicians immobilize OARs to avoid further risks and uncertainties while ensuring treatment plans deploy as intended. Currently, a variety of spacers utilize immobilization during RT. These include intraoral stents to displace and immobilize the tongue and oral anatomy, along with immobilization masks (212); omental slings or pouches fixed to the abdominal wall via sutures while separating the stomach and bowels (187); multiple synthetic implants designed to fill empty cavities and ensure the bowels are immobilized (167); and immobilization cradles that further secure the patient on the treatment couch (31, 79). Standard immobilization techniques for ablation therapies like MWA, RFA, or CA involve using probe manipulation by directly inserting one or more probes to displace and stabilize OARs. Hydrodissection can immobilize organs by injecting fluid within potential spaces, however, gravity and movement often cause the fluid to shift, necessitating additional injections.

### 4.2 Resource availability

A clinic’s ability to offer options for TOD depends on essential resources, including spacer materials, tools, and protocols, as well as the presence of “champion” users willing to implement these procedures in their clinical practice. In locoregional ablation therapy, hydrodissection is a well-established technique for separating OARs during ablation therapies, valued for its simplicity and cost-effectiveness, despite its limited organ immobilization capabilities. RT presents a broader array of TOD techniques for HNC, GI, and GU cancers, including intraoral stents, hydrogels, balloons, and prostheses. This diversity, however, complicates consensus and reproducibility across clinical centers due to varying techniques, materials, protocols, and levels of expertise. Despite technological advancements, the widespread clinical adoption of TOD remains limited, particularly in RT settings, due to a lack of standardization and clinical evidence. While Tables 1, 2 highlight a greater number of clinical studies on TOD in RT compared to locoregional ablation therapies, the existing literature remains limited, especially for cancer types beyond prostate cancer.

### 4.3 Materials and design

Researchers have explored a wide range of materials and devices for TOD, frequently adapting tools from other medical fields like cosmetic surgery, patient positioning, and cataract procedures. Material density is a crucial yet often overlooked factor when considering TOD spacers for RT (177). The material density affects how materials attenuate photons, electrons, and other particles. Water-equivalent materials with densities near 0 Hounsfield units (HU) are ideal for accurate dose calculations (234, 235), while densities ranging from 0 to 500 HU can offer visibility in CT (‘radiopacity’), with minimal dose scattering and imaging artifacts (236).

Unlike RT, locoregional ablation therapies can use low- and high-density materials to reduce thermal conductivity, minimizing damage to surrounding tissues. For hydrodissection, fluids like 5% glucose (non-conductive and well-tolerated), saline (provides thermal protection but can spread heat during RFA, though studies suggest it can be protective), and sterile water (non-conductive but may cause fluid shifts) have been considered (25, 237, 238). The choice of fluid depends on the application and careful risk-benefit analysis. Furthermore, the atomic composition of spacer materials complicates CT imaging, as different materials with similar densities or X-ray attenuation may have distinct compositions. This variation in composition leads to ambiguity in material identification and affects the accuracy of TOD techniques and treatment planning.

Lastly, biocompatibility, tolerance, biodegradability, and stability are key concerns for TOD techniques, as materials can cause patient discomfort and pain (14). The use of biologic mesh in complex hernia repairs highlights this, with high morbidity and recurrence rates despite an initial promise (239–242). Additionally, bioprosthetic materials are significantly more expensive than synthetic alternatives, often lacking reimbursement and costing up to ten times more, with price variations among manufacturers (180). The ideal material for TOD should balance efficacy, safety, and cost-effectiveness. Ogino (2013) suggests that water-equivalent materials, which enable accurate dose distribution and function as spacers before resorption, would be optimal (177). However, further research is needed to develop materials that meet these criteria while addressing the challenges of artificial materials in clinical settings.

### 4.4 Placement, verification, and analysis

The success of a TOD technique relies not only on choosing appropriate spacer materials but also on the safe and precise placement of spacers. Following spacer placement, verification methods are crucial for confirming adequate organ separation, utilizing various imaging modalities such as CT/CBCT, MRI, and US. In addition to visual inspection, quantitative metrics are vital for assessing the effectiveness of spacers, including, but not limited to, surface distance measurements, geometrical analysis, and *in silico* simulations (i.e., dose-volume histograms (DVH) and thermal data generated from RT treatment plans and microwave ablation simulation models, respectively) (see Table 3).

**TABLE 3:** Overview of supporting methods for TOD: spacer placement and immobilization, verification methods and safety measures, and evaluation metrics.

Technique	Description
Spacer placement	
Open surgery	An open surgical approach allowing implantation of synthetic or biologic mesh spacers, or procedures involving the patient’s native tissues. Examples include implantation of AlloDerm™ or Vicryl mesh, and open surgical techniques such as omental flap or ovarian transposition procedures.
Endoscopic or percutaneous placement	Minimally invasive techniques using a needle, catheter, or small incisions to position spacers without full surgical exposure. Examples include deploying inflatable balloons or the percutaneous insertion of ablation probes for organ displacement or immobilization.
Oral, ureteral, or rectal insertion	Insertion of spacers through oral, ureteral, or rectal passages. Examples include intraoral stents for oropharyngeal cancer treatment (with iterative adjustments for optimal fit and comfort), as well as ureteral and rectal stents and bladder distention devices, which help protect against adverse effects of RT for abdominal and pelvic malignancies.
Injection	Injection of fluid or gel-like materials to temporarily separate the tumor from OARs. Common approaches include transperineal, transrectal, and transabdominal routes, depending on the anatomical location of OARs. Numerous studies highlight that proper hydrodissection before hydrogel spacer placement reduces complications and improves placement accuracy (see Table 1).
Verification and safety measures	
Imaging guidance	CT: use of CT imaging to verify spacer placement and assess organ separation distances. Contrast-enhanced CT with iodinated contrast improves delineation of vascular structures and tissue boundaries. Examples include standard CT scans, contrast-enhanced CT, or cone-beam CT integrated into treatment systems for routine verification during planning and treatment.
	MRI: use of MRI to obtain high-contrast images for verifying spacer position and delineating soft tissue interfaces. Contrast-enhanced MRI with gadolinium-based agents further distinguishes between tissues. Examples include using MRI to visualize the achieved volume of injected hydrogel spacers.
	Ultrasound (US): use of US imaging to confirm the extent of organ displacement and the position of spacers. Examples include transrectal US (TRUS) during hydrodissection or hydrogel spacer deployment.
Direction visualization	Used in open or laparoscopic procedures to identify/preserve critical structures, ensure hemostasis, and minimize trauma.
Insertion precautions	Pre-aspiration and careful, incremental device or needle advancement to avoid vascular or adjacent organ injury; frequent position verification.
Hydrodissection	Saline or other fluid to dissect tissues before spacer injection; not required for all spacer types (if required).
Post-placement monitoring	Monitoring for acute or delayed complications (e.g., infection, bleeding, pain, rare risks of anaphylaxis, embolism, rectal wall injury).
Evaluation metrics	
Surface-to-surface distance measurements	Measuring the minimum distance between the tumor and adjacent OARs from imaging data. This can be done manually by scrolling through 2D CT slices or via automated algorithms (e.g., pairwise Euclidean distance or K-nearest neighbors). Volumetric analysis may also be used to quantify the extent of organ displacement.
DVH and thermal distribution data	Evaluation of spacers and their impact on patient treatment outcomes by analyzing dose plans (in RT) or thermal simulation profiles (in thermal ablation). Examples include using DVH in RT to assess dose sparing effects, typically employing dose-response models such as Tumor Control Probability (TCP) and Normal Tissue Complication Probability (NTCP) that relate DVH metrics to clinical outcomes.
	For MWA procedures, current clinical workflows rely on vendor-provided data and clinician experience to determine ablation margins by selecting optimal power (watts) and ablation time. Retrospective studies simulate thermal distribution using computational solvers that model electromagnetic wave propagation and heat transfer, incorporating temperature-dependent tissue properties (e.g., conductivity, permittivity).
Spacer scoring	Spacer Quality Score (SQS): this is an aggregated score combining prostate-rectal interspace measurements at multiple anatomic points to yield an overall assessment of spacer adequacy (higher SQS = greater, more symmetric separation and better outcomes). Currently, SQS has been primarily validated with hydrogel-based spacers in prostate cancer patients receiving RT (243, 244).
	Symmetry scoring: this score assesses symmetry of spacer placement on multiple axial slices, relative to the prostate midline. Fully symmetric placement across measured levels scores optimally; increasing asymmetry reduces the score. This symmetry scoring has been validated with hydrogel-based spacers and balloon spacers in prostate cancer patients receiving RT (245, 246).

## 5 Opportunities and future directions

**FIGURE 8:**
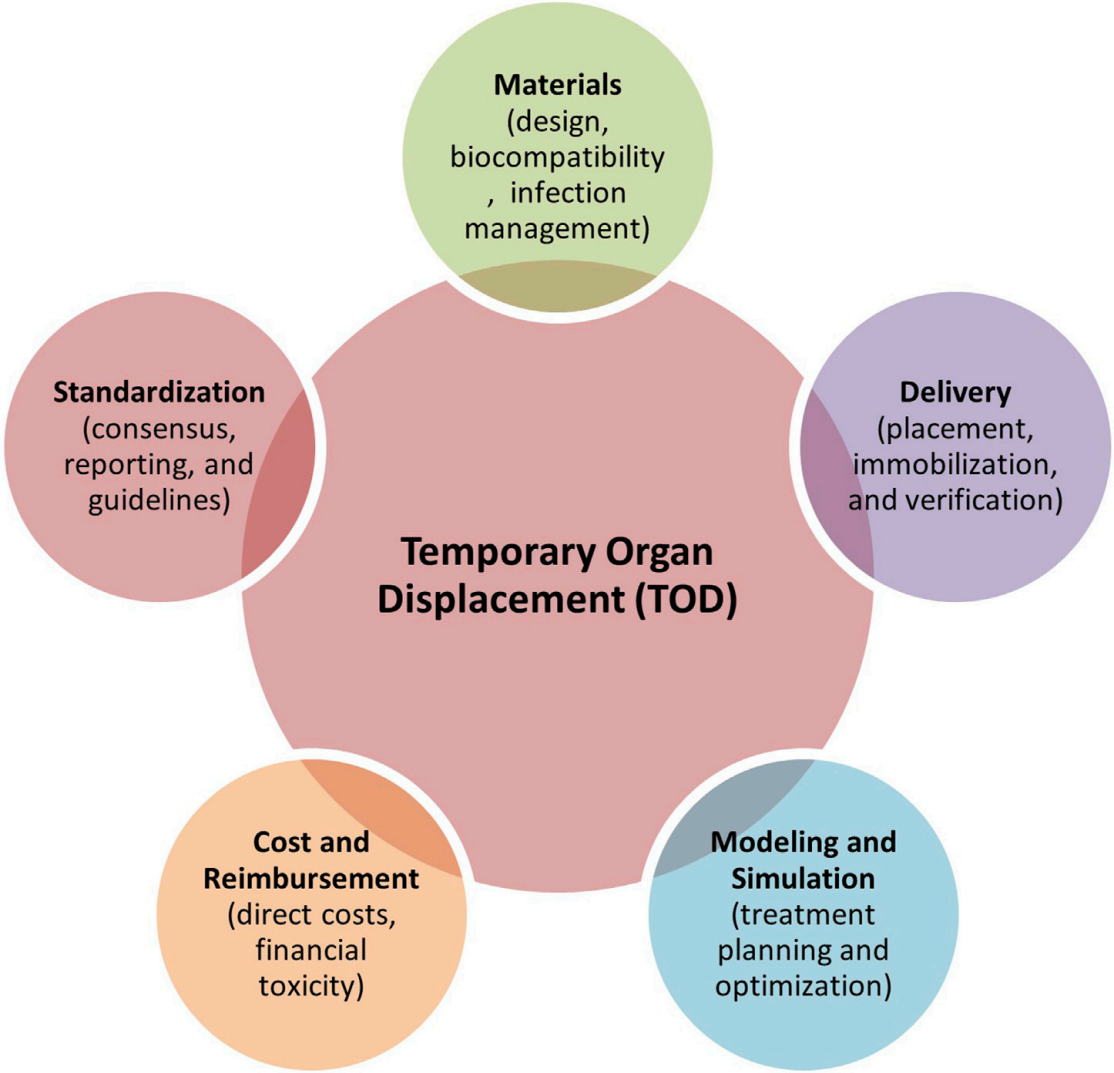
Five key areas requiring further development towards better clinical adoption of TOD techniques in cancer therapies.

Based on the current literature, TOD technologies remain limited due to awareness of the benefits of using spacers in the clinic, their limited availability, differences in the users’ technical expertise, heterogeneous patient anatomy, and time-intensive workflows (i.e., simulation time and clinical implementation in patients). Haynes et al. (1997) state that the balance between patient treatment outcomes and QOL remains a therapeutic challenge for cancer treatment and, therefore, requires evidence-based treatment approaches (247). A review of the existing literature reveals a predominance of retrospective cohort studies and case reports, with relatively few randomized controlled trials and Phase II/III studies, limiting the strength of current evidence. Many studies rely on non-randomized, single-arm designs, which, while informative, may not provide definitive conclusions on treatment efficacy (see Tables 1, 2). Several key areas need more focus to enhance the role of TOD techniques in cancer management, which are discussed below (see Figure 8).

### 5.1 Materials

Minimizing the risk of infection associated with introducing TOD spacers is crucial. This necessitates the development of improved sterile techniques, novel antimicrobial materials, and meticulous post-operative monitoring protocols. As highlighted earlier, the importance of material density and atomic composition in RT warrants further investigation to minimize dose perturbations and imaging artifacts when deploying spacers, necessitating quality control and assurance. Despite promising outcomes in using biologic spacers for RT, the ongoing risks associated with biologic mesh in hernia repair, as discussed by Brinas et al. (2018), must be carefully considered, and long-term follow-up studies are needed to assess the safety and efficacy of biologic spacers in oncologic applications (242).

### 5.2 Standardization

The lack of transparency and reproducibility issues alluded to by Ghaffari et al. (2020) regarding surgical and interventional procedures highlight the need for standardized reporting and rigorous quality control measures (161). Significant variability exists in the materials used for spacers, with ongoing debates regarding biocompatibility, density, contrast, cost, and manufacturing processes. There is no definitive consensus regarding which TOD technique is most appropriate for specific cancer therapies. Further research is needed to establish evidence-based guidelines for matching TOD techniques to specific tumor locations, sizes, and histological subtypes, considering RT and percutaneous ablation modalities. Standardized reporting guidelines are needed to address the correct and consistent reporting of variables and outcomes for reproducibility. This should include detailed descriptions of the spacer material (e.q., physical density, composition, manufacturer details), placement technique (e.q., open surgery, endoscopic/percutaneous, intraoral/ureteral/rectal insertion, or injection), imaging protocols (e.q., CT/CBCT, MRI, or US), metrics for analysis (e.q., measured separation distances between the tumor and the primary OAR, before and after spacer insertion), and any adverse events associated to spacer use. A thorough discussion of the limitations and potential biases of each study is also essential (161, 248).

### 5.3 Modeling and simulation

Current clinical trials rarely leverage biomechanical, radiation, or thermal modeling to optimize patient-specific treatment plans, particularly in spacer-mediated therapies, where variables like spacer material viscoelasticity, geometric conformity, and dynamic tissue interactions are critical for ensuring stable therapy delivery (161). Integrating these computational models into treatment workflows could significantly improve the safety and efficacy of targeted spacer delivery by validating spacer performance under thermal or mechanical stress (e.g., preventing displacement during RT) and simulating dose distribution to confirm spacer-mediated shielding of OARs. Future studies should prioritize multi-physics simulations that unify thermal diffusion, tissue biomechanics, and radiation dosimetry-for instance, finite element modeling to predict how hydrogel spacers alter dosimetry in prostate cancer or machine learning-enhanced thermal ablation simulations to optimize spacer conductivity for preventing bowel perforation. Combining machine learning with physics-based models could enable real-time adaptive planning, such as recalibrating radiation fields if intraoperative imaging detects spacer deformation or predicting spacer resorption rates to synchronize bioresorbable material degradation with fractionated treatment schedules. These advancements would not only maximize tumor control (e.g., enabling dose escalation) but also address risks, such as biomechanical displacement and unnecessary tissue damage.

### 5.4 Cost and reimbursement

As noted by Ogino (2013) the cost and reimbursement of procedures involving TOD vary widely across different countries, potentially limiting access to these interventions. Efforts are needed to advocate for equitable reimbursement policies that recognize the clinical value of TOD (177). Direct costs including specialized materials, operative time, and perioperative care must be weighed against indirect benefits such as reduced hospital stays, fewer complications, and improved QOL. Financial toxicity remains another significant concern in cancer therapies, as the inflated costs associated with the adoption of TOD may create barriers for patients and healthcare systems (249, 250). Addressing these economic challenges through policy reforms and cost-effectiveness studies is crucial to ensuring broader accessibility and equitable adoption of TOD techniques.

### 5.5 Delivery

Consensus towards the proper delivery method of TOD spacers remains unclear, with significant concerns regarding associated risks and complications for open surgical spacer placements as well as minimally invasive spacer injections. Image-guided percutaneous or endoscopic placements are increasingly preferred to enhance precision and minimize patient morbidity. While percutaneous approaches involving hydrogel spacers in RT are generally considered safer, they require precise injection within confined anatomical spaces to minimize fluid displacement (as observed in hydrodissection cases in IR), since inadequate placement can lead to ineffective organ separation and consequently result in dose toxicities (142). Although rare, they can also result in serious adverse events, including prostatitis and septic shock, underscoring the need for vigilant post-procedural monitoring, careful patient selection, and procedural expertise (251). The emergence of actively reversible NASHA^®^ spacers such as Barrigel™ (Teleflex, US) represents a significant advancement in addressing these clinical concerns (141) and is currently undergoing a large-scale, multi-center trial (NCT06496256) across the US, Australia, and Spain for PPRT patients. Recent clinical experience has demonstrated that patients with rectal wall infiltration from Barrigel™ spacers, though are uncommon, were successfully managed and reversed using targeted hyaluronidase injection (140). This reversibility feature provides a crucial safety mechanism potentially transforming the risk-benefit profile of hydrogel spacer procedures in both RT and IR applications.

To further support the evaluation of spacers and their effectiveness, the scoring methodology introduced by Fischer-Valuck et al. (2017) utilizing symmetry-based measurements relative to the prostate midline has proven clinically useful towards characterizing the impact of hydrogel-based spacers on rectal doses (245). Charas et al. (2024 ASCO Annual Meeting, Chicago, US) expanded this scoring approach to include balloon spacers (BioProtect Balloon implant™ System, BioProtect, Israel), demonstrating improved symmetry and lower rectal doses compared to gel spacers (246). Other methods, such as the spacer quality score (SQS) developed by Grossman et al. (2023), emphasize the amount of separation and spatial relationship between the spacer and rectum (244). The SQS was externally validated in a cohort of prostate cancer patients receiving SBRT (n = 30), confirming clinical feasibility, while also highlighting the need for improvements to account for the disproportionate impact of a single poorly separated region on overall spacer adequacy (243).

Current practices are increasingly looking into implementing stereotactic guided procedures in IR, and we see this naturally translate into its use for TOD spacers since both utilize complementary image-guided methodologies and share common expertise within IR protocols. In RT, similar trends begin to form using existing treatment planning platforms and computational modeling to simulate and identify optimal TOD spacer placements (252–254). Emerging technologies, including robotic-assisted navigation, augmented reality platforms, and stereotactic guidance systems, provide opportunities to further refine the delivery of TOD spacers, maximizing therapeutic efficacy while reducing risks to OARs (255, 256). One advantage of these integrated approaches is the ability to potentially achieve real-time visualization and targeting precision that enables interventional radiologists to literally see through the patient during procedures, while maintaining the reproducible accuracy.

### 5.6 Smart spacers

The advent of advanced materials offers exciting possibilities for creating ‘smart’ spacers with enhanced functionality. Drawing upon the principles of 3D printing and computer-aided design (CAD), we can tailor the properties of meta-biomaterials to meet the specific needs of individual patients (257). For instance, spacers made from stimuli-responsive materials for contraction in response to external stimuli, such as US, could dynamically adjust organ separation during RT or locoregional ablation therapies (258). Similarly, closed-loop feedback balloon spacers, which integrate sensors for real-time monitoring of pressure or received dose, offer the potential for controlled dose perturbations (259). Additionally, engineered drug-eluting spacers that incorporate therapeutic agents can deliver more localized chemotherapy, anti-inflammatory agents, or other medications, reducing the risk of dose-related toxicities and thermal damage while protecting OARs (260, 261). In addition, 3D printed balloon spacers modified with high-atomic number (Z) materials have shown potential for intraoral and rectal organ displacement and protection in rats, though the adverse effects of radiation scattering from spacers with high atomic materials remain unexplored (262). Integrating these ‘smart’ materials into spacer workflows represents a step forward in personalized medicine, offering the potential to enhance the safety and efficacy of RT and locoregional ablation therapies.

### 5.7 Patient-centric design of spacers

Our review of spacer studies reveals a diverse range of techniques, devices, and clinical outcomes, highlighting that despite significant technological advances, a “one-size-fits-all” spacer for cancer patients remains elusive. The clinical implementation of spacers is determined on a case-by-case basis, with efforts directed toward addressing accuracy and reproducibility. Notably, *in silico* studies such as predicting the minimum spacer thickness required for definitive RT with carbon ions and photons for pelvic tumors (263) as well as analyzing the comparative dosimetric impacts of a thicker omentum spacer for abdominal and pelvic tumors in carbon-ion, proton, and photon RT (264), have provided valuable insights into optimizing spacer use. Additionally, the development of cooling devices aimed at minimizing oral mucositis (265–269) and preventing skin burns during RFA of superficial paraspinal masses underscores the innovative approaches being explored to mitigate treatment-related toxicities (2).

Despite these advances, several challenges remain. Maintaining long-term stability is critical, as some spacers may migrate or degrade over time, potentially compromising organ separation and the delivery of the planned therapy. Moreover, patient-specific anatomical variability necessitates individualized approaches to spacer placement, and further advancements in imaging modalities and processing techniques are required to enhance the visualization of both spacers and surrounding tissues. Future research should focus on developing novel spacer materials that offer improved biocompatibility, stability, and visibility on imaging. In parallel, integrating machine learning algorithms into treatment planning holds promise for automating spacer placement, predicting spacer behavior, and optimizing treatment outcomes. The overarching goal is to develop and implement TOD techniques that are safe and effective and tailored to each patient’s unique needs.

### 5.8 Digital twins

Recently, the National Academies of Sciences, Engineering, and Medicine published a white paper defining a DT as a collection of virtual information constructs replicating the structure, context, and behavior of a natural, engineered, or social system. The virtual model is continuously updated with data from its physical counterpart, can predict future states, and informs decisions that create value. The two-way interaction between the virtual model and the physical entity is central to the DT concept (270).

**FIGURE 9:**
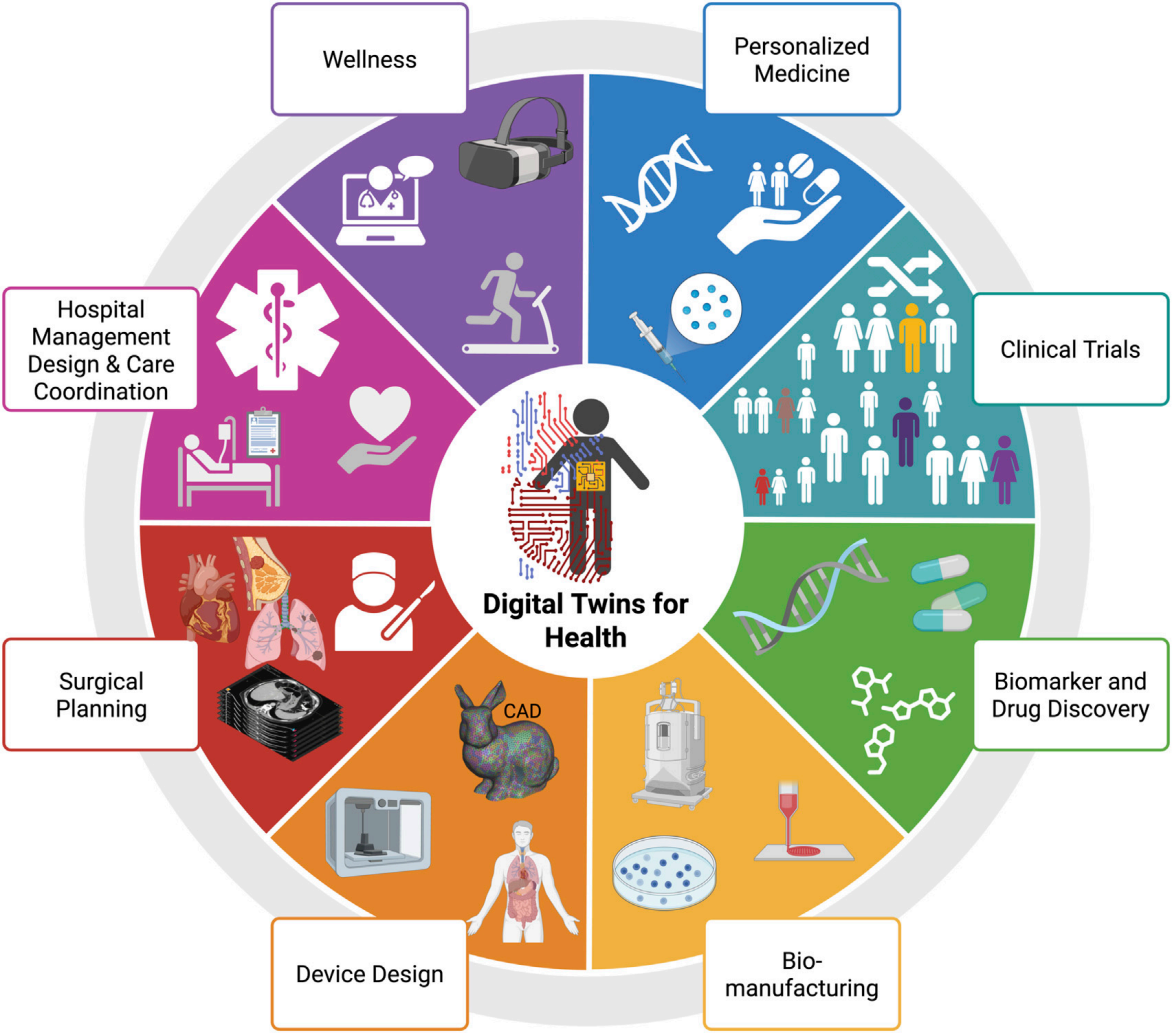
Primary applications of digital twins (DT) in healthcare. Adapted with permission from “Main applications of digital twins for health” by Evangelia Katsoulakis, Qi Wang, Huanmei Wu, Leili Shahriyari, Richard Fletcher, Jinwei Liu, Luke Achenie, Hongfang Liu, Pamela Jackson, Ying Xiao, Tanveer Syeda-Mahmood, Richard Tuli and Jun Deng, licensed under CC BY 4.0. Created with BioRender.com.

Within healthcare, DT is categorized into eight key areas: wellness, personalized medicine, clinical trials, biomarker and drug discovery, biomanufacturing, device design, surgical planning, and hospital management design and care coordination (see Figure 9). By integrating TOD into DT models that leverage real-time imaging data, virtual environments, and simulations, clinicians gain enhanced accuracy, guidance, and support to maximize patient treatment outcomes while minimizing complications. This integrated approach enables more precise and personalized cancer therapies, improving efficacy and safety. This section explores key concepts in RT and locoregional ablation therapies, focusing on the interface between TOD and DT.

#### 5.8.1 Planning, simulation, and clinical decision making in RT

Early concepts of DT towards minimizing RT damage have long been implemented in the areas of medical physics. Treatment planning systems (TPS) have utilized the synergy across 3D organ models and Monte Carlo models to maximize tumor dose prescription, accounting for predefined OAR constraints (6, 272). Later on, advanced image-guidance, tracking, and deformable image registration methods were introduced to accurately account for movements and breathing (10, 273–275). Other DT-based applications in oncology involve the integration of mechanistic modeling, which provides key insights into cancer dynamics, treatment, and patient outcomes (276–278).

**FIGURE 10:**
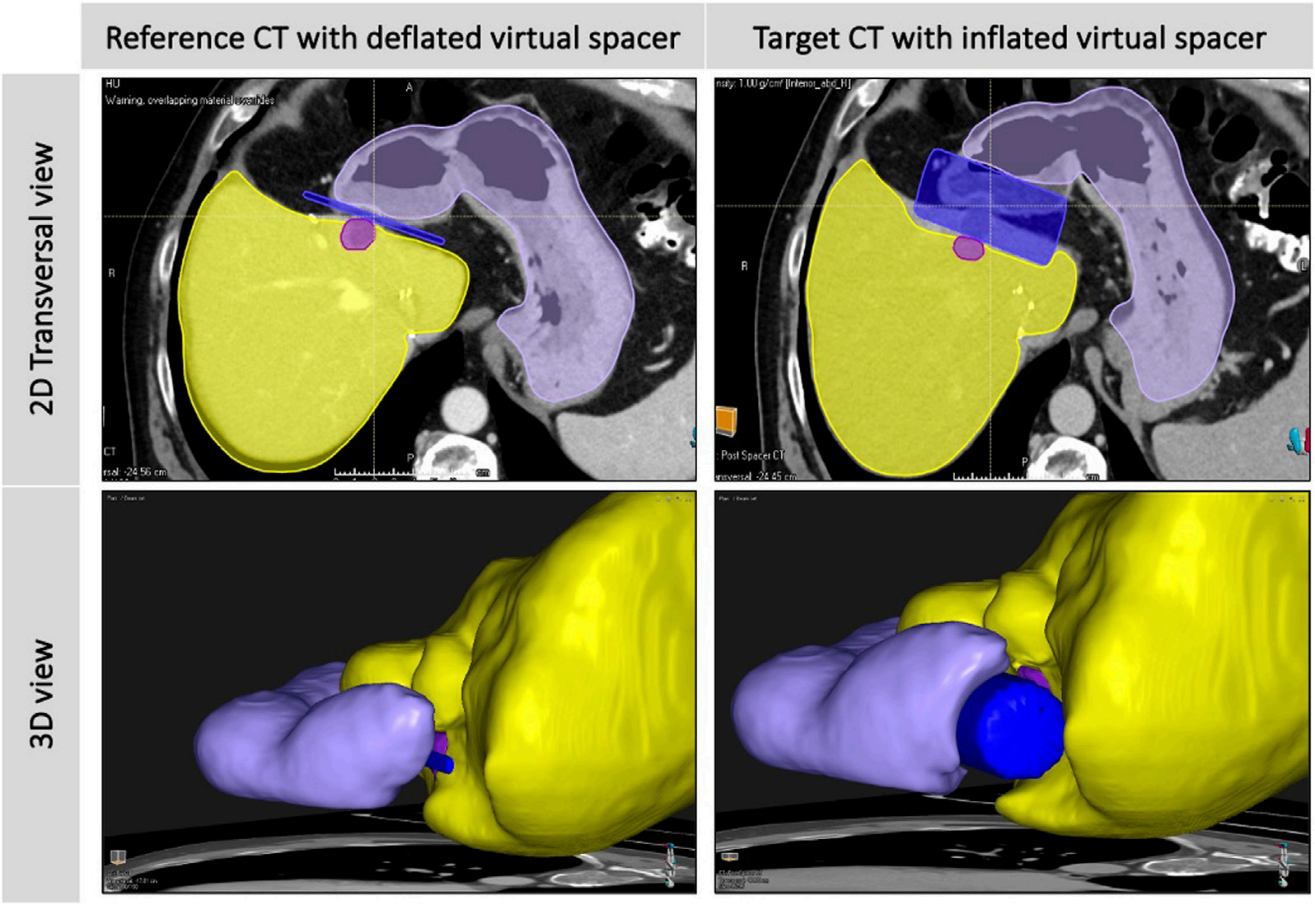
A diagram showing the *in silico* deformation of liver (yellow), stomach (purple), and tumor (light purple) structures, after simulating the inflation of a virtual cylindrical spacer (blue) from reference to target CT using a biomechanical deformable registration tool (Morfeus, RayStation V12.0, Raysearch Laboratories AB, Sweden).

Building on the importance of spacer placement and shape for improved RT dosimetry and planning (303, 304), recently developed DT-based spacer planning methods have emerged as an innovative solution, leveraging existing TPS and the finite element method (FEM) to understand spacer placement and its dosimetric impact better (see Figure 10). Kawaguchi et al. (2021) conducted a treatment planning study to investigate the dosimetric effects of a virtual spacer placed between the pancreas and surrounding OARs, highlighting the importance of accurate spacer placement to minimize non-target tissue coverage (279). Similarly, Yamada et al. (2019) compared the dosimetric impacts of a greater omentum spacer across carbon-ion, proton, and photon RT plans, showing notable dose-reduction effects of the virtual omental spacer on the GI tract (i.e., bowel, colon, rectum) (264). Furthermore, the same group developed a multi-regression model to predict the minimum spacer thickness required, that is, the minimum distance between the tumor and the GI tract, that allows for the delivery of definitive doses within the dose limits (D2% <77 Gy RBE) (263). Van Wijk et al. (2017) demonstrated the combination of a multi-regression model and an image deformation model using a unidirectional field not only to predict geometrical changes of the rectum and prostate in the presence of a virtual implantable rectum spacer but also to predict the resulting GI toxicity (280).

More recently, Hooshangnejad et al. (2021) demonstrated a personalized approach by developing an FEM-based spacer simulation algorithm, FEMOSSA, to produce realistic deformations of the rectum and the prostate wall in the presence of a virtual HS (253). This algorithm, in combination with a Bayesian multi-regression model, has proven to be highly valuable as a potential clinical decision support tool for patients with pancreatic tumors receiving duodenal spacer placement procedures (252, 281). More recently, a robotic arm was developed to precisely control the placement of a ‘smart’ balloon applicator for brachytherapy and intraoperative RT. This innovation aims to enhance targeted tumor irradiation while effectively shielding OARs (SkinCure Oncology LLC, IL, United States) (259, 282, 283).

#### 5.8.2 Planning, simulation, and clinical decision making in IR

Several simulation platforms have been proposed and adapted for planning thermal ablation procedures. More specifically, multi-physics solvers to compute electromagnetic power, temperature distribution, and specific absorption rates were investigated for MWA applications (284–287). Additionally, mathematical models involving isotherm contours, thermal effective doses, and the Arrhenius model have added value towards more accurate quantification of cell death during thermal ablation procedures (288, 289). Zhang et al. (2019) highlighted key computational approaches currently utilized for planning thermal ablation procedures encompassing the segmentation of anatomical structures from imaging scans, identifying the tumor’s centroid, and defining feasible skin entry zones through ‘hard’ or user-defined constraints. Subsequently, ‘Soft’ constraints are implemented to refine needle trajectory, and an optimization framework determines the ideal needle path. Despite significant algorithmic progress, clinical adoption remains limited primarily due to the lack of clinical evidence, highlighting a gap between research and practice (290).

A multi-modal approach utilizing a combination of DT-based technologies can possibly bridge this gap. Technologies such as deformable imaging methods, robotics, virtual reality, 3D printing, and stereotactic navigation systems have been integrated into IR planning protocols to enhance workflows and potentially improve treatment outcomes (8, 256, 291–296). More recently, Paolucci et al. (2025) highlight that automated, software-based workflows for ablation confirmation have transformed the assessment of technical success in percutaneous thermal ablation of malignant liver tumors. Conventional methods to measure minimal ablative margins (MAM) involves the use of anatomic landmarks, a method limited by operator bias and poor reproducibility. State-of-the-art software solutions now enable standardized, quantitative MAM measurement by integrating advanced imaging techniques, segmentation, and registration, many of which are enhanced by artificial intelligence. These digital tools provide rapid, accurate feedback during procedures, allowing for immediate re-ablation if margins are insufficient, and supporting improved local tumor control (255).

Commercial systems such as Ethicon’s NeuWave and Medtronic’s Emprint offer distinct MWA planning approaches (9). NeuWave integrates with picture archiving and communication systems (PACS) for lesion identification and antenna targeting but lacks a visual display of the predicted ablation zone, necessitating manual margin reference. In contrast, Emprint employs organ-specific workflows to automatically extract imaging data and calculate ablation times based on *in vivo* data. In addition, CAScination’s CAS-One IR integrates optical stereotactic navigation to enhance needle targeting (297–299). Innovations in stereotactic CT guidance with CAS-One IR have improved precision, reduced repositioning, and minimized complications such as bleeding and tumor dissemination (300, 301). Recent CT-based applications have successfully treated challenging lesions, as seen in a breast cancer liver metastasis in segment I that was precisely ablated with no recurrence after 18 months. While MWA offers advantages over RFA, such as resistance to the heat sink effect and the ability to create larger ablation zones, these benefits must be carefully managed to avoid damage to critical structures. Integrating advanced navigation systems like CAS-One IR in the clinic is a crucial step toward optimizing ablation outcomes and minimizing procedural risks (302).

## 6 Summary

In this review, we synthesized the considerable progress in the development of TOD techniques and devices utilized for RT and locoregional ablation therapies. Here, we provide a comprehensive classification of TOD based on key technical and clinical parameters. This structured approach is vital for addressing challenges such as the lack of standardized guidelines, variability in technical proficiency, and complex clinical workflows. Ultimately, this classification aims to strengthen clinical outcomes, foster broader adoption, and pave the way for the next-generation of more effective TOD techniques and better-tolerated cancer therapies. This review culminates in several key findings and future directions:

• From non-invasive and indirect TOD techniques such as deep inspiration breath-hold and advanced immobilization devices to more invasive and direct techniques like intraoral stents, injection, and surgical spacer placements, the field has experienced a remarkable diversification of organ displacement strategies. These innovations have enhanced the precision of radiation delivery and locoregional ablation therapies and significantly decreased treatment-related morbidities. The impact of these innovations extends beyond immediate clinical outcomes. By enabling more aggressive treatment regimens while reducing side effects, these techniques can improve long-term survival rates, enhance QOL for cancer survivors, and reduce the economic burden of managing treatment-related complications.
• Standardization of TOD techniques in clinical practice remains an evolving priority. While significant progress has been made in refining organ displacement strategies, universally accepted guidelines and protocols for TOD implementation remain limited. This absence of standardization risks compromising reproducibility, outcome comparability, and broader integration across cancer treatment centers. Addressing these challenges is critical to maximize clinical benefits, ensure high-quality care, enable informed treatment planning, as well as facilitate the development of robust clinical trials. Standardizing TOD techniques will empower clinicians to better interpret treatment effectiveness and safety, supporting evidence-based decisions and paving the way for broader adaptation in diverse healthcare settings.
• Integrating TOD techniques with emerging technologies promises to enhance and personalize cancer treatments further. Smart spacers utilizing meta-materials offer exciting possibilities for enhancing the precision of RT and thermal ablation therapies. These innovative devices can automatically tune to optimize treatment delivery while minimizing damage to healthy tissues. These advanced technologies can improve treatment outcomes, combined with artificial intelligence-driven treatment planning, real-time imaging, and adaptive therapies. The ongoing research in this field, including using sophisticated simulations to optimize spacer design and placement, underscores the medical community’s commitment to innovation.

## Glossary

3D: Three-dimensional
3DCRT: 3D conformal radiation therapy
ABC: Active breathing coordinator
ADT: Androgen deprivation therapy
BMS: Biologic mesh spacer
BT: Brachytherapy
CA: Cryoablation
CCA: Cholangiocarcinoma
CAD: Computer-aided design
CBCT: Cone beam computed tomography
CRC: Colorectal carcinoma
CRLM: Colorectal liver metastasis
CT: Computed tomography
D5W: 5% dextrose in water
DIBH: Deep inspiration breath hold
DT: Digital twin
DVH: Dose to volume histogram
EBRT: External beam radiation therapy
ePTFE: Expanded polytetrafluoroethylene
FB: Free breathing
FEM: Finite-element method
GCT: Giant cell tumor
GI: Gastrointestinal
GU: Genitourinary
HA: Hyaluronic acid
HCC: Hepatocellular carcinoma
HDR: High-dose rate
HNC: Head and neck
HS: Hydrogel spacer
HU: Hounsfield units
ICRU: International commission on radiation units and measurements
IG-IMRT: Image-guided, intensity-modulated radiotherapy
IMBT: Intensity-modulated brachytherapy
IMPT: Intensity-modulated proton therapy
IMRT: Intensity-modulated radiation therapy
IORT: Intraoperative radiotherapy
IR: Interventional radiology
LAD: Left anterior descending artery
LDR: Low-dose rate
MRI: Magnetic resonance imaging
MWA: Microwave ablation
NSCLC: Non-small cell lung cancer
NSGCT: Non-seminomatous germ cell tumors
NTCP: Normal tissue complication probability
OAR: Organs-at-risk
OS: Overall survival
PACS: Picture archiving and communication system
PBRT: Proton beam radiation therapy
PD: Pancreaticoduodenal
PEG: Polyethylene glycol
PGA: Polyglycolic acid
PTC: Papillary thyroid carcinoma
PTMC: Papillary thyroid microcarcinoma
QOL: Quality of life
RCC: Renal cell carcinoma
RFA: Radiofrequency ablation
RIOM: Radiation-induced oral mucositis
RT: Radiation therapy
SBRT: Stereotactic body radiation therapy
TCC: Transitional cell carcinoma
TRUS: Transrectal Ultrasound
TOD: Temporary organ displacement
TPS: Treatment planning system
TCP: Tumor control probability
US: Ultrasound
VMAT: Volumetric arc radiation therapy
